# Dysregulated overexpression of Sox9 induces fibroblast activation in pulmonary fibrosis

**DOI:** 10.1172/jci.insight.152503

**Published:** 2021-10-22

**Authors:** Prathibha R. Gajjala, Rajesh K. Kasam, Divyalakshmi Soundararajan, Debora Sinner, Steven K. Huang, Anil G. Jegga, Satish K. Madala

**Affiliations:** 1 Department of Pediatrics, College of Medicine, University of Cincinnati, Cincinnati, Ohio, USA.; 2 Division of Pulmonary Medicine and; 3 Divisions of Neonatology and Pulmonary Biology, Perinatal Institute, Cincinnati Children’s Hospital Medical Center, Cincinnati, Ohio, USA.; 4 Division of Pulmonary and Critical Care Medicine, Department of Internal Medicine, University of Michigan Medical School, Ann Arbor, Michigan, USA.; 5 Division of Biomedical Informatics, Cincinnati Children’s Hospital Medical Center, Cincinnati, Ohio, USA.

**Keywords:** Pulmonology, Apoptosis, Collagens, Fibrosis

## Abstract

Idiopathic pulmonary fibrosis (IPF) is a fatal fibrotic lung disease associated with unremitting fibroblast activation including fibroblast-to-myofibroblast transformation (FMT), migration, resistance to apoptotic clearance, and excessive deposition of extracellular matrix (ECM) proteins in the distal lung parenchyma. Aberrant activation of lung-developmental pathways is associated with severe fibrotic lung disease; however, the mechanisms through which these pathways activate fibroblasts in IPF remain unclear. Sry-box transcription factor 9 (Sox9) is a member of the high-mobility group box family of DNA-binding transcription factors that are selectively expressed by epithelial cell progenitors to modulate branching morphogenesis during lung development. We demonstrate that Sox9 is upregulated via MAPK/PI3K-dependent signaling and by the transcription factor Wilms’ tumor 1 in distal lung-resident fibroblasts in IPF. Mechanistically, using fibroblast activation assays, we demonstrate that Sox9 functions as a positive regulator of FMT, migration, survival, and ECM production. Importantly, our in vivo studies demonstrate that fibroblast-specific deletion of Sox9 is sufficient to attenuate collagen deposition and improve lung function during TGF-**α**–induced pulmonary fibrosis. Using a mouse model of bleomycin-induced pulmonary fibrosis, we show that myofibroblast-specific Sox9 overexpression augments fibroblast activation and pulmonary fibrosis. Thus, Sox9 functions as a profibrotic transcription factor in activating fibroblasts, illustrating the potential utility of targeting Sox9 in IPF treatment.

## Graphical abstract

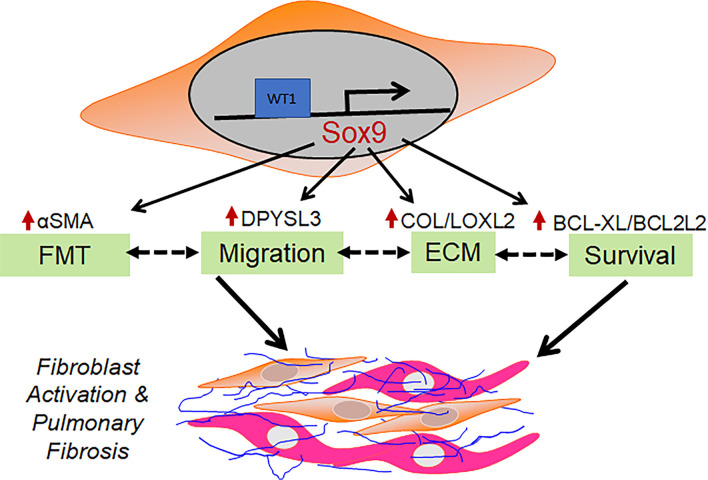

## Introduction

Pulmonary fibrosis is the final common pathway of several lung diseases caused by dysregulated healing responses to chronic lung injury. Fibrosis is often associated with fibroproliferation, survival, and excessive deposition of the extracellular matrix (ECM) proteins, leading to progressive lung parenchyma scarring (1, 2). Idiopathic pulmonary fibrosis (IPF) is perhaps the most severe and enigmatic form of interstitial lung disease, and recent epidemiological studies suggest that the prevalence of the disease is increasing in the United States and globally. The mortality rate of IPF is increasing every year as well, and the disease is one of the leading causes of death among the aging population (2). The survival rate after diagnosis is typically 3 to 5 years, which is partly due to a lack of effective treatments or cure. However, the major impediment to developing new therapies is the lack of knowledge regarding mechanisms underlying fibroblast activation in IPF. Therefore, it is essential to characterize and understand these underlying molecular mechanisms in order to develop new treatments that attenuate fibroblast activation in IPF.

Transcription factors play a critical role in orchestrating cell-specific gene networks that support the early stages of lung development. The expression of these transcription factors is typically suppressed in fully developed adult lungs, preventing cellular overgrowth and lung dysfunction. Studies using single-cell transcriptomic analysis of lung cells suggest the existence of a cell-specific expression pattern and aberrant reactivation of these developmental transcription factors in a cell-specific manner in IPF (3, 4). In particular, frequent injury of and damage to epithelial cells have been shown to reactivate several profibrotic transcription factors in lung cells; reactivation results in dysregulated repair and regeneration in IPF and in several chronic mouse models of severe fibrotic lung disease. Recent studies from our lab and others have demonstrated the reactivation of Wilms’ tumor 1 (WT1) in both fibroblasts and mesothelial cells in IPF lungs. In contrast, during lung development, WT1 expression is limited to mesothelial cells, and the lack of such expression is lethal to mice. Furthermore, our next-generation RNA-Seq studies using WT1-positive fibroblasts identified several important transcriptional regulators, such as Sry-box transcription factor 9 (*Sox9*), that are epithelial cell specific and involved in lung development (5). Sox9 is a transcription factor that belongs to Sry-related high-mobility group box–containing proteins. Insights into Sox9 expression and function have been primarily obtained from studies of embryonic organ development; chondrogenesis in the trachea, branching morphogenesis in the lung, and male gonad development are examples (6–8). In developing lungs, Sox9 is highly expressed in the distal tips of the branching epithelium. Sox9 expression is downregulated by the onset of terminal differentiation into type I and type II epithelial cells and is restricted to only few airway epithelial cells (5). The overexpression or deletion of Sox9 leads to branching defects and affects proliferation, differentiation, and ECM production (6, 9).

Although the role of Sox9 in the epithelium during lung development is well documented, Sox9 expression in the distal fibroblasts of the lung and its potential role in fibroblast activation have never been explored in the context of pulmonary fibrosis to our knowledge. In the present study, we evaluated whether Sox9 is upregulated in fibroblasts and the possible pathogenic role of Sox9, and its target genes, in fibroblast activation and pulmonary fibrosis. Our study demonstrates that Sox9 upregulation in fibroblasts contributes to pulmonary fibrosis and thus provides a therapeutic target for IPF.

## Results

### SOX9 is upregulated in distal lung fibroblasts in IPF.

**Figure 1:**
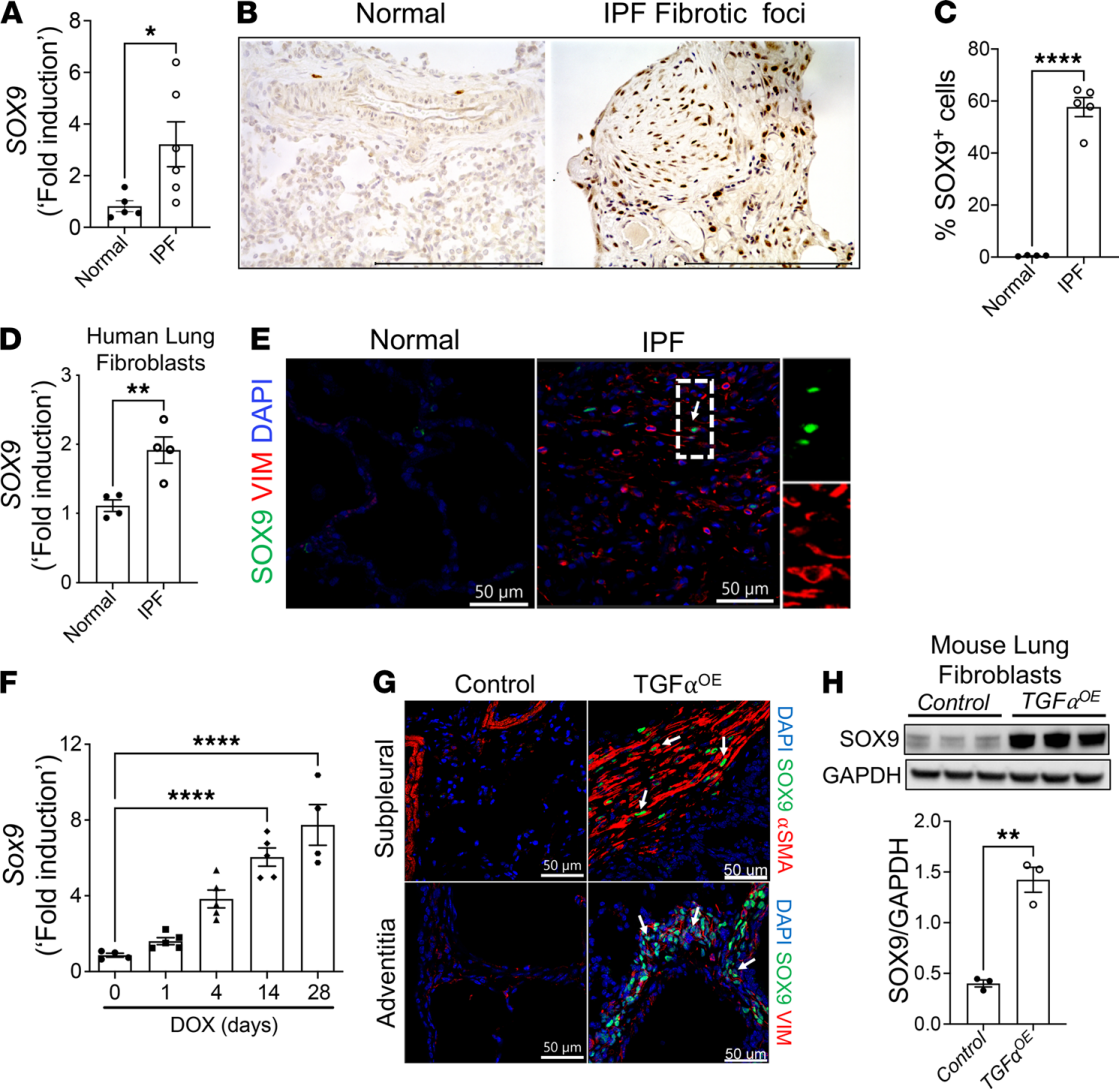
SOX9 is upregulated in the distal lung fibroblasts during the pathogenesis of pulmonary fibrosis. (**A**) Quantification of *SOX9* transcripts from the total lung RNA of healthy individuals and patients with IPF by real-time PCR (RT-PCR). (*P < 0.05; *n* = 5–6/group; Student’s 2-tailed *t* test.) (**B**) Immunostaining was performed with the anti-SOX9 antibody on lung sections of normal individuals and patients with IPF. Representative images were obtained at 40× original magnification. Scale bar: 150 μm (*n* = 5/group). (**C**) Quantification of SOX9-positive cells normalized to the total lung cells in images of lung sections from normal individuals and patients with IPF was performed using a BZ-X analyzer. (****P < 0.00005; *n* = 5/group; Student’s 2-tailed *t* test.) (**D**) Quantification of *SOX9* transcripts in primary lung fibroblasts grown from normal and IPF lungs by RT-PCR. (**P < 0.005; *n* = 4/group; Student’s 2-tailed *t* test.) (**E**) Confocal microscopy images of lung sections from normal individuals and patients with IPF stained with anti-SOX9 (green color) and anti-vimentin (red color) antibodies and nuclei stained with DAPI (blue color). Cells positive for both SOX9 and vimentin are highlighted with white arrows. Images obtained at 60× original magnification. Scale bar: 50 μm (*n* = 5/group). (**F**) Quantification of *Sox9* transcripts in the total lung transcripts of *TGF*α*OE* mice treated with Dox for 0, 1, 4, 14, and 28 days (****P < 0.00005; *n* = 4–5/group; 1-way ANOVA). (**G**) Confocal images of lung sections from control and *TGF*α*OE* mice stained with anti-SOX9 (green color) and anti-ACTA2 (red color) or anti-vimentin (red color) antibodies and nuclei stained with DAPI (blue color). Scale bar: 50 μm (*n* = 5/group). (**H**) Primary lung-resident fibroblasts isolated from lung cultures of control and *TGFα^OE^* mice treated with Dox for 4 weeks were immunoblotted with antibodies against SOX9 and GAPDH. SOX9 protein levels were normalized to GAPDH and are shown as fold induced change using a bar graph (**P < 0.005, *n* = 3/group; Student’s 2-tailed *t* test).

To identify the kinetics of SOX9 expression, we measured *SOX9* transcripts during the embryonic and postnatal stages of lung development and observed that *Sox9* RNA levels were the highest in the early lung (E14.5), and subsequently declined before birth, with the lowest RNA levels observed in the adult lungs (Supplemental Figure 1A; supplemental material available online with this article; https://doi.org/10.1172/jci.insight.152503DS1). Immunofluorescence staining of SOX9 in the lung sections of alpha-actin-2 (ACTA2) reporter mice supported earlier findings that SOX9 is selectively expressed by distal epithelial progenitor cells; however, there was limited or no staining observed in the ACTA2-positive mesenchymal cells of developing lungs at E15.5 (Supplemental Figure 1B). Furthermore, measurement of *SOX9* transcript levels revealed a significant increase in the total lung transcripts of *SOX9* in individuals with IPF compared with those in healthy controls (Figure 1A). Immunohistochemistry revealed a potentially previously unreported finding that SOX9 is predominantly localized to the nuclei of spindle-shaped cells in the distal fibrotic lesions including fibrotic foci and thickened subpleura of the lung, which are the predominant pathological features of IPF (Figure 1B and Supplemental Figure 2A). Quantification of the number of SOX9-positive cells and total cells in these fibrotic lesions indicated a significant increase in the percentage of SOX9-positive cells in IPF compared with that in healthy controls (Figure 1C). To show that SOX9 is upregulated in IPF fibroblasts, we measured *SOX9* transcripts in the distal lung fibroblasts isolated from IPF and healthy donor lungs. Indeed, we observed a marked increase in the *SOX9* transcripts in the total transcripts of fibroblasts isolated from IPF lungs, compared with that in fibroblasts from normal lungs (Figure 1D). Analysis of IPF and normal lung sections coimmunostained with antibodies against SOX9 and vimentin showed a marked increase in the number of SOX9 and vimentin dual-positive cells in IPF lungs compared with those in normal lungs (Figure 1E). Similarly, analysis of fibroblasts from distal lung cultures showed a significant increase in the number of SOX9-positive fibroblasts in IPF (Supplemental Figure 2, B and C).

### Sox9 is upregulated in a mouse model of TGF-α–induced pulmonary fibrosis.

To determine whether SOX9 is upregulated during TGF-α–induced pulmonary fibrosis, we measured the *Sox9* levels in the total lung transcripts of TGF-α–overexpressing (*TGF*α*OE*) mice that developed severe fibrotic lung disease by 28 days after doxycycline (Dox) treatment (10). *Sox9* transcript levels in the lungs of Dox-treated *TGF*α*OE* mice were the highest at day 28 compared with those at day 0 (Figure 1F). Coimmunostaining analyses revealed an increase in the dual-positive cells for ACTA2 and SOX9, or vimentin and SOX9, in the fibrotic lesions of *TGF^OE^* mice compared with control mice treated with Dox for 6 weeks (Figure 1G). We also observed a significant increase in SOX9 protein levels in lysates of the distal lung fibroblasts isolated from *TGF*α*OE* mice compared with those from control mice treated with Dox for 4 weeks (Figure 1H). These results suggest that Sox9 is upregulated in the distal lung fibroblasts of IPF and a mouse model of TGF-α–induced pulmonary fibrosis.

### The TGFα/WT1 axis is involved in the upregulation of SOX9 in fibroblasts.

**Figure 2:**
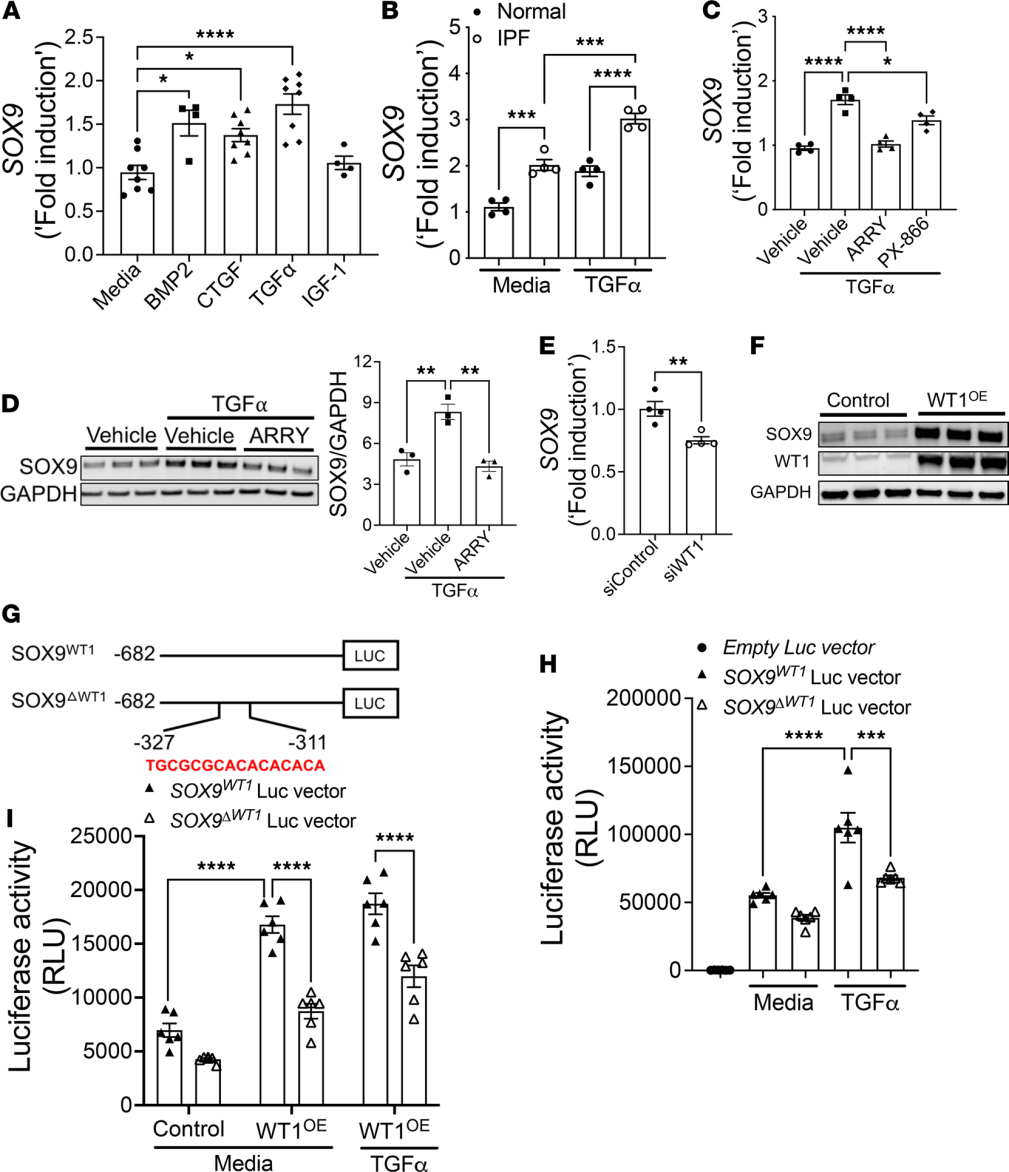
The TGF-α/WT1 axis induces SOX9 in the distal lung fibroblasts. (**A**) *SOX9* transcripts were measured by RT-PCR in normal lung fibroblasts treated with media or BMP2 (200 ng/mL), CTGF (50 ng/mL), TGF-α (100 ng/mL), and IGF1 (100 ng/mL) for 16 hours. (****P < 0.00005, *P < 0.05; *n* = 4–8/group; 1-way ANOVA.) (**B**) Quantification of *SOX9* transcripts by RT-PCR in normal and IPF lung fibroblasts treated with media or TGF-α (100 ng/mL) for 16 hours. (****P < 0.00005, ***P < 0.0005; *n* = 4; 1-way ANOVA.) (**C**) Quantification of *SOX9* transcripts by RT-PCR in IPF fibroblasts treated with vehicle, MEK inhibitor (ARRY, 0.1 μM), or PI3K inhibitor (PX-866, 0.1 μM) in the presence and absence of TGF-α (100 ng/mL) for 16 hours. (****P < 0.00005, *P < 0.05; *n* = 4; 1-way ANOVA.) (**D**) IPF fibroblasts were treated with vehicle or MEK inhibitor (ARRY, 1 μM) in the presence and absence of TGF-α (100 ng/mL) for 48 hours and immunoblotted with antibodies against SOX9 and GAPDH. SOX9 protein levels were normalized to GAPDH and are shown as fold induced change using a bar graph. (**P < 0.005; *n* = 3; Student’s 2-tailed *t* test.) (**E**) IPF lung fibroblasts were transiently transfected with control or WT1 siRNA for 72 hours, and *SOX9* transcripts were quantified using RT-PCR. (**P < 0.005; *n* = 4; Student’s 2-tailed *t* test.) (**F**) Normal lung fibroblasts were transduced with control or WT1-adenoviral particles for 72 hours, and cell lysates were immunoblotted with antibodies against SOX9, WT1, and GAPDH. (**G**) Schemata show a human *SOX9* promoter (*Sox9^WT1^*) clone or the WT1 binding site with the *SOX9* promoter clone deleted (*Sox9*^Δ^*WT1*). (**H**) Luciferase activity was measured in HEK293 cells that were transiently transfected with empty, *SOX9^WT1^*, or *SOX9*^Δ^*WT1* promoter plasmids and cells treated with media or TGF-α (100 ng/mL) for 16 hours (****P < 0.00005, ***P < 0.0005; *n* = 6; 1-way ANOVA). (**I**) Luciferase activity was measured in HEK293 cells cotransfected with WT1 overexpression plasmid and *SOX9^WT1^* or *SOX9*^Δ^*WT1* promoter-luciferase plasmids for 48 hours and then treated with either media or TGF-α (100 ng/mL) for another 16 hours. (****P < 0.00005; *n* = 6, 1-way ANOVA).

To determine the potential effects of multiple profibrotic growth factors on SOX9 expression, we treated distal human lung fibroblasts (HLFs) with multiple growth factors, including TGF-α, bone morphogenic protein 2 (BMP2), connective tissue growth factor (CTGF), and insulin growth factor 1 (IGF1). *SOX9* levels were elevated in HLFs treated with TGF-α, BMP2, and CTGF; however, there was limited or no effect of IGF1 on *SOX9* levels (Figure 2A). We next studied the effects of TGF-α on SOX9 expression in fibroblasts from IPF and normal lungs. We observed that *SOX9* levels were high in both normal and IPF fibroblasts treated with TGF-α compared with those in media-treated fibroblasts (Figure 2B). To determine the signaling pathway involved in TGF-α–induced SOX9 expression, we treated IPF fibroblasts with inhibitors of MEK (ARRY) and PI3K (PX-866) in the presence and absence of TGF-α. As expected, TGF-α treatment resulted in a notable increase in the phosphorylation of Erk1/2 at 30 minutes compared with that in media-treated fibroblasts (Supplemental Figure 3, A–C). This increase in TGF-α–induced phosphorylated (p-) Erk1/2 was significantly attenuated in IPF fibroblasts treated with ARRY compared with that in vehicle-treated controls (Supplemental Figure 3, A–C). Notably, we observed a significant decrease in TGF-α–induced SOX9 expression in IPF fibroblasts treated with ARRY (Figure 2C). Although basal levels of p-AKT were decreased in the presence of TGF-α compared with vehicle treatment, further inhibition of p-AKT with PX-866 resulted in a significant reduction in *SOX9* levels in IPF fibroblasts treated with TGF-α (Figure 2C). Next, we quantified the changes in SOX9 protein levels in IPF fibroblasts treated with TGF-α in the presence and absence of ARRY and observed a significant reduction in TGF-α–induced SOX9 protein in IPF fibroblasts (Figure 2D). Recent studies from our laboratory have shown that WT1 is upregulated in activated fibroblasts, and it coincides with the increase in profibrotic gene networks, including *Sox9*, during TGF-α–induced pulmonary fibrosis (3, 4). To test whether WT1 is a positive regulator of SOX9, we knocked down WT1 in distal lung fibroblasts isolated from IPF lungs. Compared with control siRNA, the knockdown of WT1 was sufficient to attenuate *SOX9* expression in IPF fibroblasts (Figure 2E and Supplemental Figure 3D). We next assessed the effect of adenovirus-mediated overexpression of WT1 on *SOX9* levels in normal HLFs. Overexpressing WT1 in fibroblasts significantly increased both transcript and protein levels of SOX9 (Figure 2F and Supplemental Figure 3, E–H). To test whether the TGF-α/WT1 axis is involved in upregulation of *Sox9*, we treated cultured lung fibroblasts from *WT1^fl/fl^* (control) and *Col1*α*2^CreER^ WT1^fl/fl^* mice with 4-hydroxy-tamoxifen in the presence and absence of TGF-α. With the loss of WT1, we observed a significant decrease in *Sox9* levels in fibroblasts treated with TGF-α (Supplemental Figure 3, I and J). To determine whether WT1 is a transcription factor that directly binds to the *SOX9* promoter, we performed a computational analysis of the promoter region of human *SOX9* and found the presence of a WT1 binding site (Figure 2G). We next generated *SOX9* promoter-driven luciferase reporter clones that either contained a WT1-binding site (*SOX9^WT1^*) or did not (*SOX9*^Δ^*WT1*) to validate the role of the TGF-α/WT1 axis in inducing SOX9 expression. We transfected HEK293 cells with either an empty plasmid containing the luciferase reporter gene or a *SOX9* promoter-driven luciferase reporter gene (*SOX9^WT1^* or *SOX9*^Δ^*WT1*) and treated cells with TGF-α to observe changes in the luciferase activity (Figure 2H). Compared with cells treated with the empty reporter plasmid, cells transfected with *SOX9* promoter-driven luciferase reporter clones showed increased luciferase activity (Figure 2H). This increase in *SOX9* promoter-driven luciferase activity was further elevated in cells treated with TGF-α compared with media-treated cells. In the presence of TGF-α, the deletion of the *WT1*-binding element in the *SOX9* promoter (*SOX9*^Δ^*WT1*) markedly decreased *SOX9* promoter activity compared with cells transfected with the *SOX9^WT1^* promoter plasmid (Figure 2H). We next overexpressed WT1 in HEK293 cells cotransfected with either the *SOX9^WT1^* or *SOX9*^Δ^*WT1* luciferase reporter plasmid. We observed a significant increase in luciferase activity in cells cotransfected with the *WT1* overexpression plasmid compared with cells transfected with the control plasmid (Figure 2I). Importantly, the observed increase in the *SOX9* promoter activity with WT1 overexpression was significantly attenuated with the deletion of the *WT1*-binding site in the *SOX9*-luciferase promoter. Together, our findings suggest that WT1 functions as a positive regulator of SOX9 by directly binding to the *SOX9* promoter in the distal lung fibroblasts during TGF-α–induced fibroblast activation and pulmonary fibrosis.

### SOX9 is a positive regulator of fibrosis-associated gene networks.

**Figure 3:**
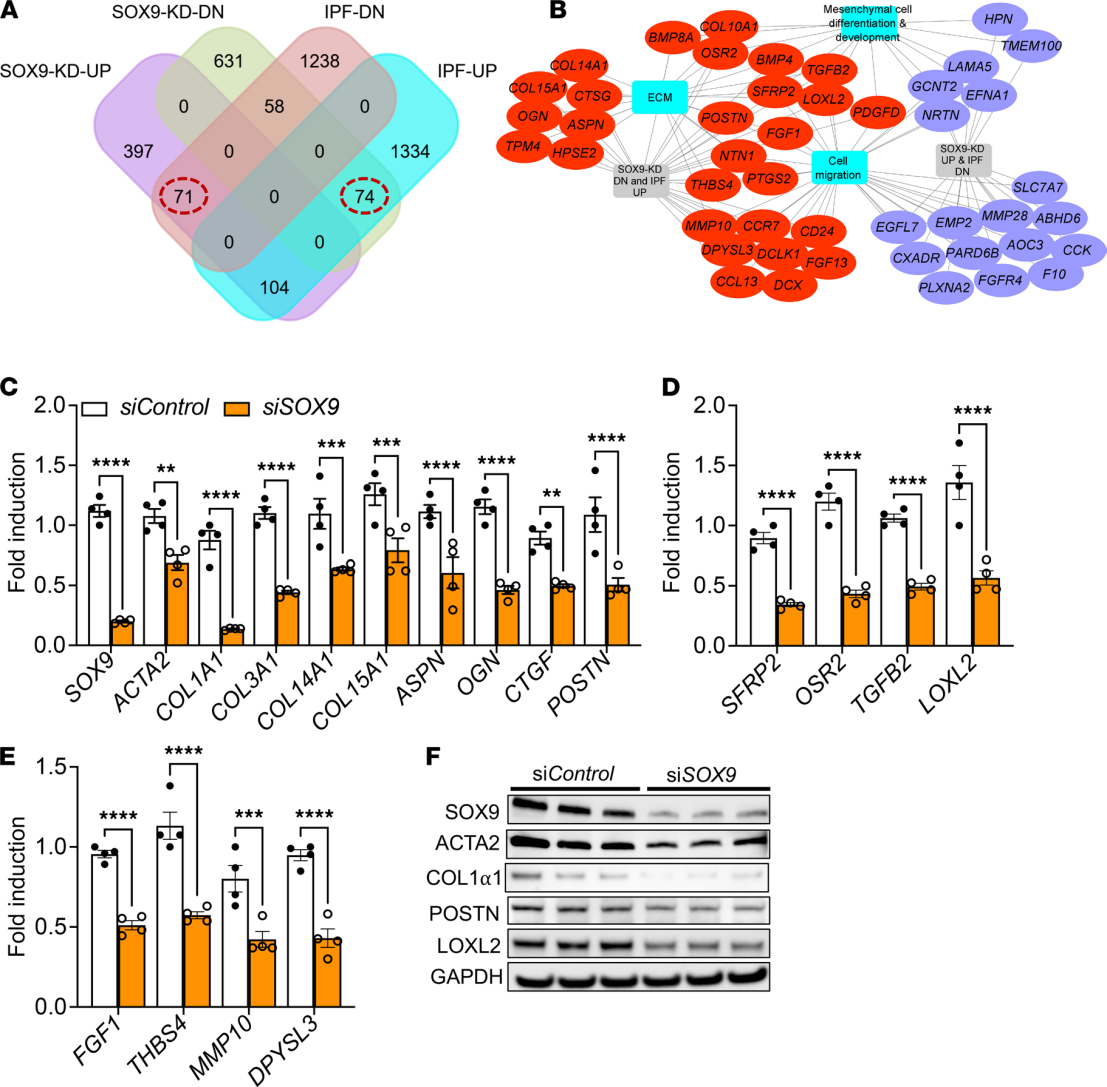
Loss of SOX9 attenuates the genes involved in fibroblast activation in IPF. (**A**) The Venn diagram shows an overlap between differentially expressed genes in SOX9-deficient IPF fibroblasts and IPF lungs. (**B**) SOX9-driven gene networks in IPF fibroblasts were analyzed using ToppFun and visualized using Cytoscape. Red and purple colored oval shapes represent SOX9-driven genes that are up- or downregulated in IPF lungs, respectively. The turquoise squares represent enriched biological processes for inversely correlated genes between SOX9-deficient fibroblasts and IPF lungs. (**C**) Quantification of ECM-related gene transcripts by RT-PCR in IPF fibroblasts treated with either control or *SOX9*-specific siRNA for 72 hours. (****P < 0.00005, ***P < 0.0005, **P < 0.005; *n* = 4; Student’s 2-tailed *t* test.) (**D**) Quantification of gene transcripts implicated in mesenchymal cell differentiation by RT-PCR in IPF fibroblasts treated with either control or *SOX9*-specific siRNA for 72 hours (****P < 0.00005; *n* = 4; Student’s 2-tailed *t* test). (**E**) Quantification of migration-associated gene transcripts by RT-PCR in IPF fibroblasts treated with either control or *SOX9*-specific siRNA for 72 hours. (****P < 0.00005, ***P < 0.0005; *n* = 4; Student’s 2-tailed *t* test.) (**F**) IPF fibroblasts were treated with either control or *SOX9*-specific siRNA for 72 hours, and cell lysates were immunoblotted with antibodies against SOX9, ACTA2, COL1α1, POSTN, LOXL2, and GAPDH.

To gain additional insights into the role of SOX9 in fibroblast activation, we assessed differentially expressed gene transcripts with the loss of SOX9. Specifically, we performed next-generation sequencing of total RNA from IPF fibroblasts treated with SOX9-specific siRNA in comparison with those treated with control siRNA for 72 hours. As shown in the heatmap, SOX9 knockdown resulted in 572 upregulated and 763 downregulated genes (≥1.5-fold change; *P* ≤ 0.05; Supplemental Figure 4A). To identify SOX9-driven profibrotic gene transcripts in IPF, we next compared differentially expressed genes in SOX9-deficient IPF fibroblasts with up- or downregulated gene transcripts in IPF lungs (National Center for Biotechnology Information Gene Expression Omnibus GSE53845; ref. 11). Our comparative analysis identified multiple differentially expressed gene transcripts that were either upregulated (74 genes) or downregulated (71 genes) by SOX9 in IPF, as shown in the Venn diagram (Figure 3A). The list of genes is shown in Supplemental Table 1. Functional enrichment analysis of negatively correlated gene sets, using the ToppFun application of the ToppGene Suite, identified several important fibroblast-specific biological processes; these genes were found to be associated with ECM production and organization, migration, and mesenchymal cell differentiation (Figure 3B and Supplemental Figure 4A). To validate the SOX9-driven profibrotic genes in IPF fibroblasts, we quantified transcripts using real-time PCR for ECM genes (*COL1A1*, *COL3A1*, *COL14A1*, *COL15A1*, and *ACTA2*) and proteoglycan-associated genes (*ASPN*, *OGN*, *POSTN*, and *CTGF*; Figure 3C). Additionally, the loss of SOX9 resulted in a significant decrease in gene transcripts associated with mesenchymal cell differentiation and development (*SFRP2*, *OSR2*, *TGFB2*, and *LOXL2*), as well as migration (*FGF1*, *THBS4*, *MMP10*, and *DPYSL3*; Figure 3, D and E). We next confirmed the knockdown effects of SOX9 on ECM protein levels through Western blot analysis of IPF fibroblasts treated with SOX9-specific siRNA compared with those treated with control siRNA for 72 hours. Consistent with changes in gene transcripts, we observed a significant decrease in ECM-associated protein levels with the loss of SOX9 in IPF fibroblasts (COL1α1, ACTA2, POSTN, and LOXL2; Figure 3F and Supplemental Figure 4B).

### SOX9 is a positive regulator of fibroblast migration, transformation, and survival.

**Figure 4:**
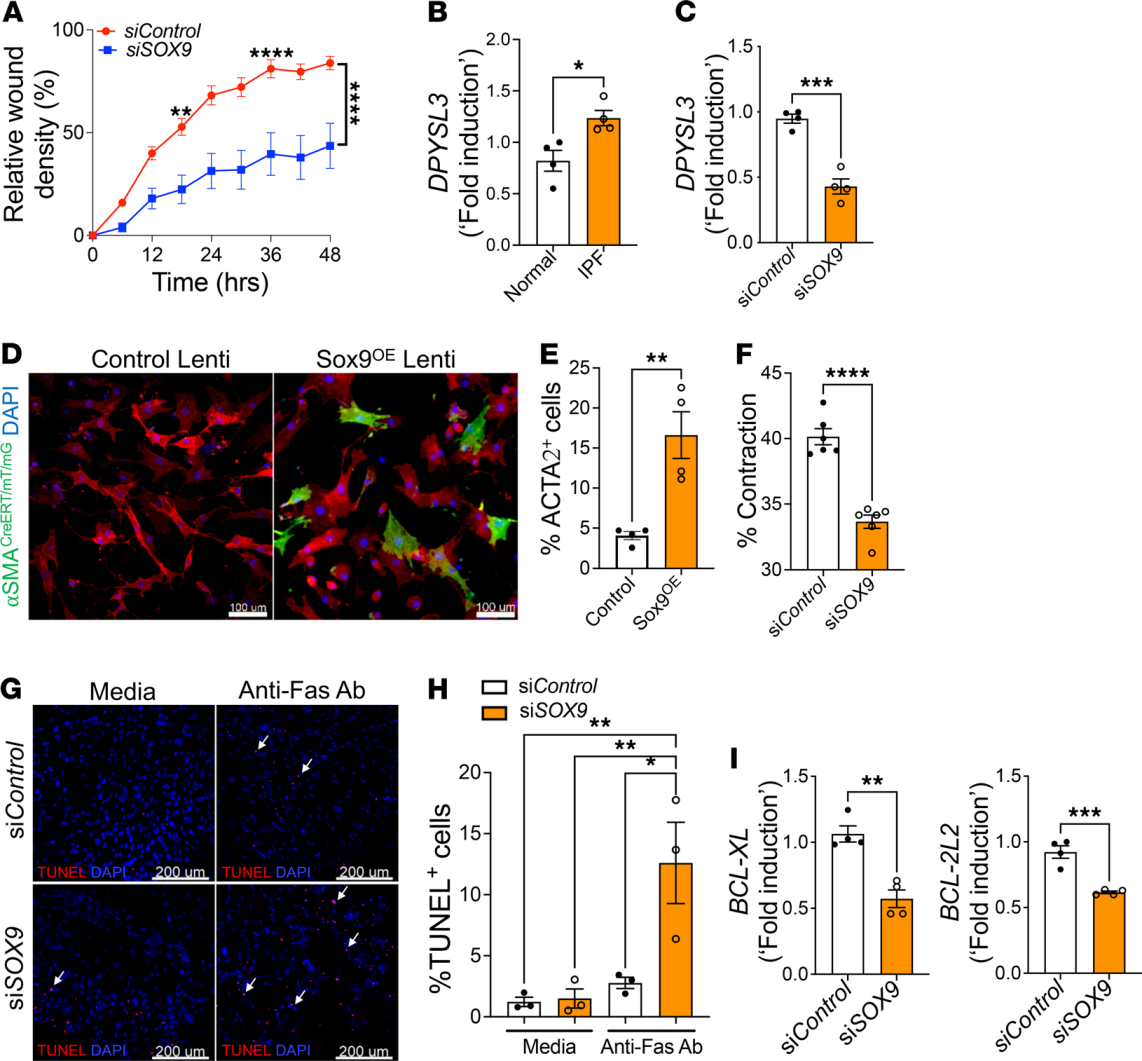
SOX9 is a positive regulator of migration, transformation, and survival of IPF fibroblasts. (**A**) Quantification of migration of IPF fibroblasts treated with either control or *SOX9* siRNA for 72 hours. (****P < 0.00005, **P < 0.005; *n* = 12; 2-way ANOVA.) (**B**) Quantification of *DPYSL3* gene transcripts in the total transcripts of IPF compared with normal lungs (*P < 0.05; *n* = 4; Student’s 2-tailed *t* test). (**C**) Quantification of *DPYSL3* gene transcripts in IPF fibroblasts treated with either control or *SOX9* siRNA for 72 hours (***P < 0.0005; *n* = 4; Student’s 2-tailed *t* test). (**D**) Fibroblasts isolated from distal lung cultures of α*SMA^CreERT^ Rosa^mTmG^* mice were transduced with either control or SOX9-overexpressing lentivirus in the presence of 4-hydroxy-tamoxifen for 72 hours. Representative confocal images were obtained at 60× original magnification. Scale bar: 100 μm (*n* = 4/group). (**E**) The number of ACTA2-positive (GFP or green color) cells in the total cells (DAPI or blue color) was quantified using MetaMorph image analysis software and presented as the percentage of ACTA2-positive cells in total fibroblasts (**P < 0.005; *n* = 4; Student’s 2-tailed *t* test). (**F**) IPF fibroblasts were treated with either control or *SOX9* siRNA for 72 hours and cultured with collagen gels to measure the changes in collagen gel contraction after 6 hours (****P < 0.00005; *n* = 6; Student’s 2-tailed *t* test). (**G**) IPF fibroblasts were treated with either control or *SOX9* siRNA for 48 hours, followed by anti-Fas treatment for another 24 hours, and cells stained to quantify the total TUNEL-positive cells. Representative confocal images were obtained at 20× original magnification. Scale bar: 200 μm. (**H**) The numbers of TUNEL-positive (red color) cells and the total cells (DAPI or blue color) were quantified using Elements image analysis software and are presented as the percentage of TUNEL-positive cells in total cells. (**P < 0.005, *P < 0.05; *n* = 3/group; 1-way ANOVA). (**I**) Quantification of antiapoptotic gene transcripts, *BCL-XL* and *BCL-2L2*, in IPF fibroblasts treated with either control or *SOX9*-specific siRNA for 72 hours (***P < 0.0005, **P < 0.005; *n* = 4; Student’s 2-tailed *t* test).

Bioinformatics analysis of Sox9-knockdown transcriptome in IPF fibroblasts suggested that SOX9 may function as a positive regulator of fibroblast migration, fibroblast-to-myofibroblast transformation (FMT), and fibroblast survival in IPF. To test whether SOX9 induces the migration of fibroblasts, we performed real-time scratch assays using IPF fibroblasts treated with control or SOX9-specific siRNA for 48 hours. We then measured the kinetics of migration. With SOX9 knocked down, IPF fibroblasts showed a significant reduction in their ability to migrate (Figure 4A and Supplemental Figure 5A). Functional enrichment analyses of SOX9-regulated genes involved in migration identified dihydropyrimidinase-like-3 (DPYSL3) as a potential downstream target of SOX9 in mediating fibroblast migration in IPF. Therefore, we assessed whether DPYSL3 is upregulated in IPF and observed a significant increase in *DPYSL3* gene transcripts in the total lung transcripts of IPF compared with those in normal lungs (Figure 4B). SOX9 knockdown significantly reduced *DPYSL3* gene transcript levels in IPF fibroblasts (Figure 4C). These results suggest that SOX9 is a positive regulator of fibroblast migration and induces migration-associated genes, including *DPYSL3*, in IPF fibroblasts. Because we observed altered ACTA2 expression with the loss or overexpression of SOX9, we also assessed whether SOX9 induces FMT. To evaluate the role of SOX9 in FMT, we used a cell-fate-mapping strategy based on the lineage-specific expression of ACTA2 in lung-resident fibroblasts isolated from *Acta2* reporter mice (α*SMA^CreERT^ Rosa^mTmG^* mice). In this model, the distal lung fibroblasts of the *Acta2* reporter mice were infected with either control or SOX9-overexpressing lentiviruses in the presence of 4-hydroxy-tamoxifen for 72 hours. Upon lentivirus-mediated overexpression of SOX9, we observed a significant increase in the number of myofibroblasts (cells shown in green), suggesting that SOX9 induces FMT (Figure 4, D and E). To determine whether SOX9 contributes to the contractile function of myofibroblasts, we treated IPF fibroblasts with control or SOX9-specific siRNA for 72 hours and seeded them into rat tail collagen gels. We observed a significant reduction in the contractility of collagen gels with the loss of SOX9 in IPF fibroblasts (Figure 4F and Supplemental Figure 4B). To determine whether SOX9 induces resistance to Fas-induced apoptosis in fibroblasts, we performed a TUNEL assay in IPF fibroblasts treated with either control or SOX9-specific siRNA for 72 hours. SOX9 knockdown in IPF fibroblasts resulted in a significant increase in TUNEL-positive fibroblasts with the treatment of anti-Fas antibody compared with control antibody (Figure 4, G and H). Therefore, we measured the changes in antiapoptotic gene transcripts and observed a significant decrease in the transcripts of antiapoptotic genes, including *BCL-XL* and *BCL-2L2*, in SOX9-deficient fibroblasts (Figure 4I). These findings suggest that SOX9 induces the antiapoptotic pathway genes responsible for enhanced survival of IPF fibroblasts. Taken together, our in vitro results establish that SOX9 upregulation in distal lung fibroblasts augments fibroblast activation, including migration, FMT, survival, and ECM production.

### Fibroblast-specific deletion of Sox9 attenuates TGF-α–induced pulmonary fibrosis in mice.

**Figure 5:**
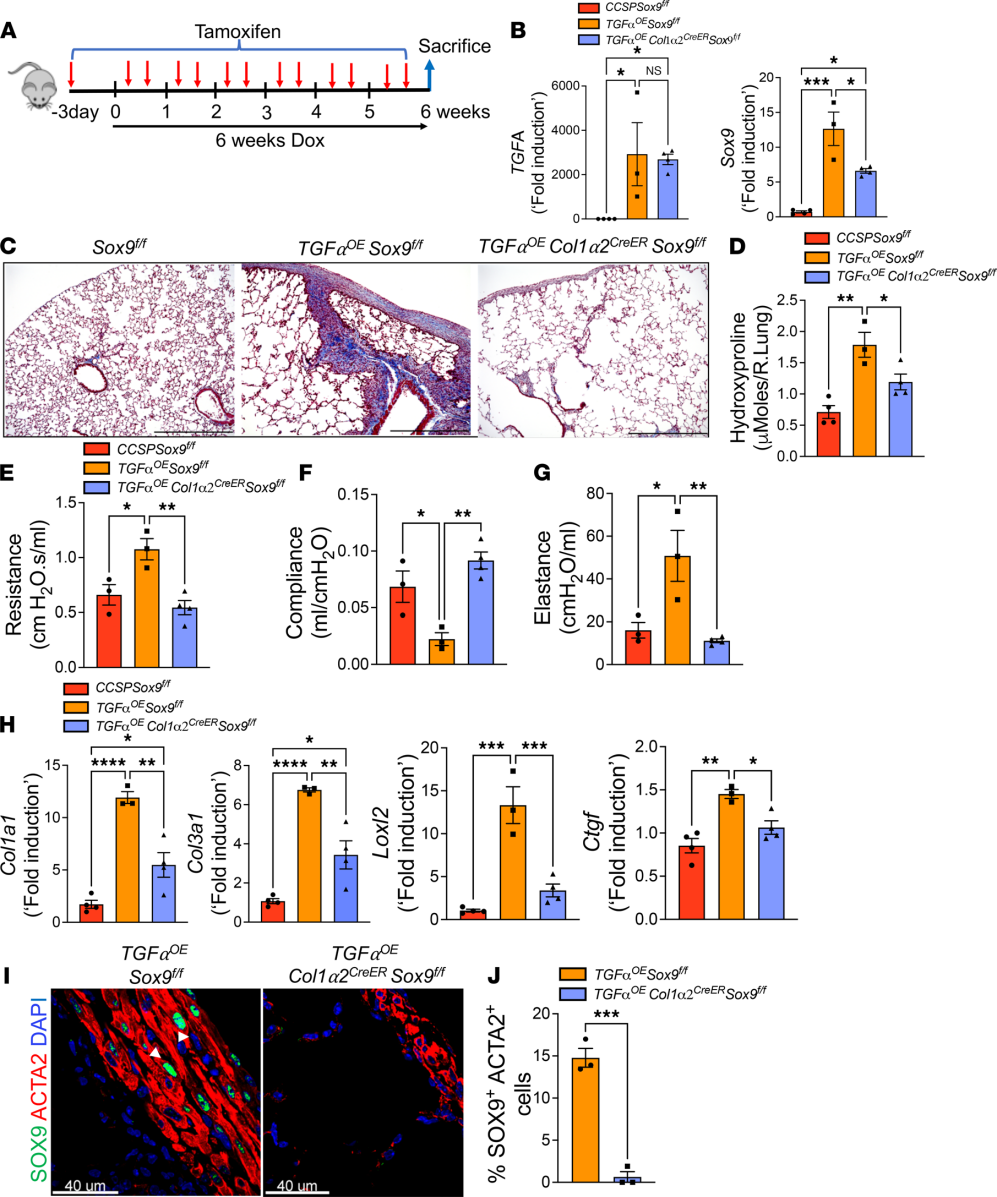
Fibroblast-specific deletion of SOX9 attenuates TGF-α–induced pulmonary fibrosis in vivo. (**A**) Representation of animal experiments performed with 3 groups of *Sox9^fl/fl^*, *TGFα^OE^ Sox9^fl/fl^*, and *TGFα^OE^ Col1α2^CreER^ Sox9^fl/fl^* mice. All mice were treated with Dox for 6 weeks in conjunction with 2 intraperitoneal tamoxifen injections per week. (**B**) Quantification of *TGFα* and *Sox9* gene transcripts in the total lungs of control and Sox9-deficient mice. (***P < 0.0005, *P < 0.05; *n* = 3–4/group; 1-way ANOVA.) (**C**) Representative images of Masson’s trichrome–stained lung sections from *CCSP Sox9^fl/fl^*, *TGFα^OE^ Sox9^fl/fl^*, and *TGFα^OE^ Col1α2^CreER^ Sox9^fl/fl^* mice. Images were captured at 10× original magnification. Scale bar: 500 μm. (**D**) Quantification of hydroxyproline levels in the right lung of mice. (**P < 0.005, *P < 0.05; *n* = 3–4/group; 1-way ANOVA.) (**E**–**G**) Quantification of lung mechanics (resistance, compliance, and elastance) among 3 groups of mice using FlexiVent (**P < 0.005, *P < 0.05; *n* = 3–4/group; 1-way ANOVA). (**H**) Quantification of *Col1a1*, *Col3a1*, *Ctgf*, and *Loxl2* gene transcripts in the total lungs of control and Sox9-deficient mice (****P < 0.00005, ***P < 0.0005, **P < 0.005, *P < 0.05; *n* = 3–4/group; 1-way ANOVA). (**I**) Lung sections of fibrosis control (*TGFα^OE^ Sox9^fl/fl^*) and Sox9-deficient (*TGFα^OE^ Col1α2^CreER^ Sox9^fl/fl^*) mice were immunostained with antibodies against SOX9 (green color) and ACTA2 (red color). Representative confocal images were obtained at 60× original magnification, and DAPI was used to stain nuclei (blue color). Scale bar: 40 μm. White arrowheads were used to highlight cells positive for both SOX9 and ACTA2. (**J**) The SOX9- and ACTA2-positive cells in the total cells were quantified using Elements image analysis software. ***P < 0.0005; *n* = 3/group.

To investigate the in vivo effects of fibroblast-specific Sox9 deletion in the pathogenesis of pulmonary fibrosis, we crossed *TGF*α*OE* mice with Sox9-floxed mice and *Col1*α*2^CreER^* mice. Through breeding, we successfully generated 3 groups of mice, including control (*CCSP Sox9^fl/fl^*) mice and *TGF*α*OE* mice containing fibroblast-specific Cre and the floxed Sox9 alleles (*TGF*α*OE Sox9^fl/fl^* mice or *TGF*α*OE Col1*α*2^CreER^ Sox9^fl/fl^* mice). To identify whether fibroblast-specific Sox9 deletion influences the susceptibility to fibrosis, all groups of mice were treated with Dox for 6 weeks and simultaneously treated with tamoxifen to delete Sox9 in fibroblasts (Figure 5A). To assess whether TGF-α expression is similar in both the *TGF*α*OE Sox9^fl/fl^* and *TGF*α*OE Col1*α*2^CreER^Sox9^fl/fl^* groups, we quantified its transcripts and found the expression to be similar in both groups (Figure 5B). To determine the efficiency of *Col1*α*2^CreER^*-driven Sox9 deletion, we measured transcripts of *Sox9* in all 3 groups of mice. As expected, we observed an increase in the *Sox9* transcript levels in Dox-treated *TGF*α*OE Sox9^fl/fl^* mice compared with *Sox9^fl/fl^* mice. Approximately 50% of *Sox9* transcripts that were elevated in the lungs of *TGF*α*OE Sox9^fl/fl^* mice were attenuated in *TGF*α*OE Col1*α*2^CreER^ Sox9^fl/fl^* mice (Figure 5B). Importantly, the reduction in *Sox9* transcripts was associated with a significant decrease in collagen staining and in the total lung hydroxyproline levels in *TGF*α*OE Col1*α*2^CreER^ Sox9^fl/fl^* mice compared with *TGF*α*OE Sox9^fl/fl^* mice (Figure 5, C and D). Consistent with changes in lung collagen levels, we observed a significant improvement in lung function parameters, including resistance, compliance, and elastance, in *TGF*α*OE Col1*α*2^CreER^ Sox9^fl/fl^* mice compared with *TGF*α*OE Sox9^fl/fl^* mice (Figure 5, E–G). Furthermore, transcripts of several ECM-associated genes, including *Col1a1*, *Col3a1*, *Ctgf*, and *Loxl2*, that were elevated in *TGF*α*OE Sox9^fl/fl^* mice compared with control mice were significantly attenuated in *TGF*α*OE Sox9^fl/fl^* mice (Figure 5H). To identify Sox9-positive myofibroblast accumulation, lung sections were immunostained with antibodies against ACTA2 and SOX9. Accumulation of SOX9-positive myofibroblasts was attenuated in fibrotic lung lesions of Dox-treated *TGF*α*OE Col1*α*2^CreER^ Sox9^fl/fl^* mice compared with *TGF*α*OE Sox9^fl/fl^* mice (Figure 5, I and J). Thus, our findings provide direct in vivo evidence that Sox9 expression in fibroblasts plays a pathogenic role during TGF-α–induced pulmonary fibrosis.

### Sox9 is required for the maintenance of fibroblast activation.

**Figure 6:**
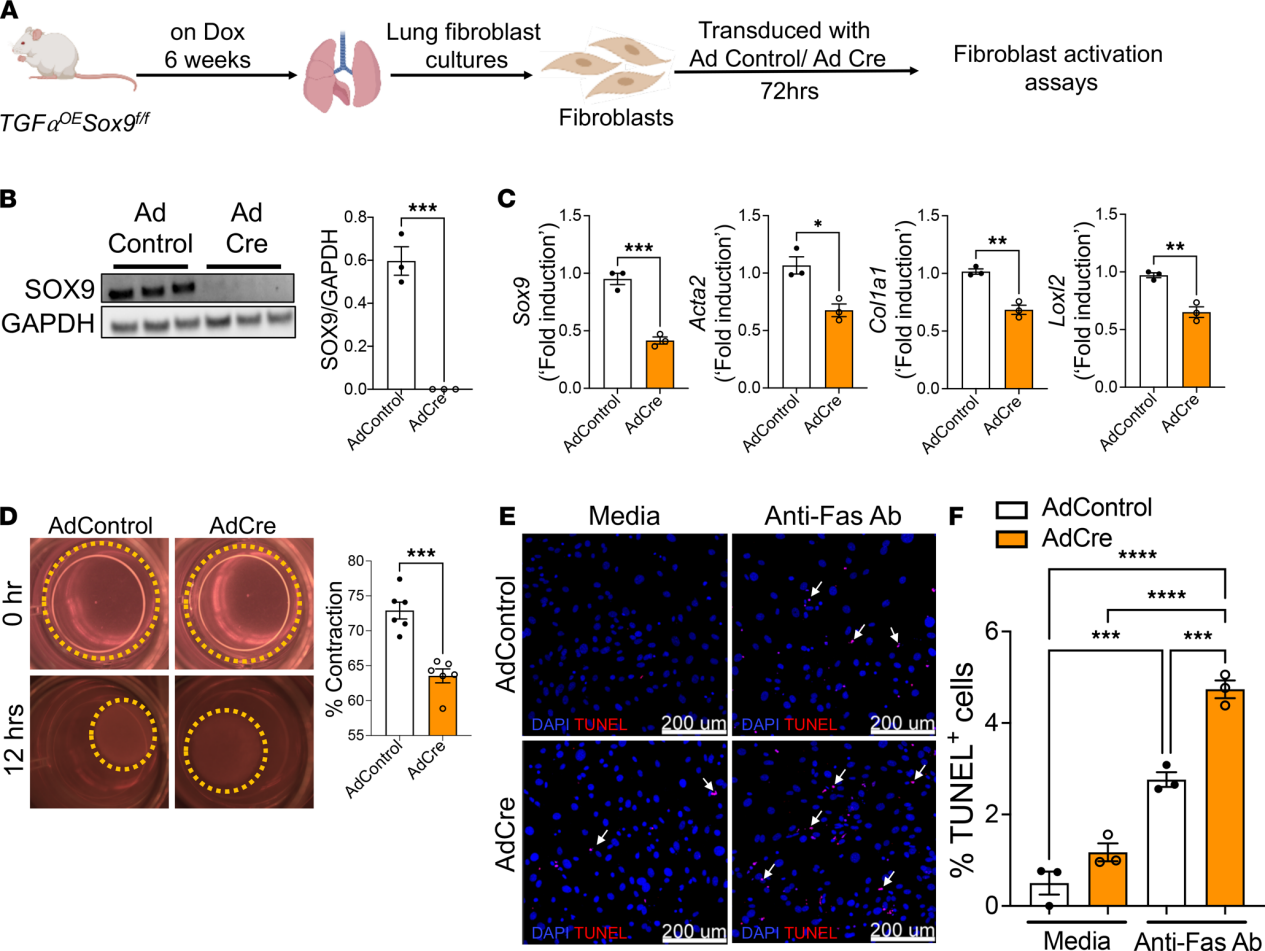
SOX9 is required for the maintenance of fibroblast activation. (**A**) Schematic representation of the workflow for isolating fibroblasts from lung cultures of *TGFα^OE^Sox9^fl/fl^* mice treated with Dox for 6 weeks. Fibroblasts were infected with either control or Cre-expressing adenovirus for 48 hours or 72 hours to delete SOX9 in activated fibroblasts. (**B**) Fibroblasts were infected with control or Cre-expressing adenovirus for 72 hours, and cell lysates were immunoblotted with antibodies against SOX9 and GAPDH. SOX9 protein levels were normalized to GAPDH and are shown as induced fold change using a bar graph (***P < 0.0005; *n* = 3, Student’s 2-tailed *t* test). (**C**) Fibroblasts were transduced with control or Cre-expressing adenovirus for 72 hours and were measured the transcripts of *Sox9*, *Acta2*, *Col1a1*, and *Loxl2* by RT-PCR. (***P < 0.0005, **P < 0.005, *P < 0.05; *n* = 3; Student’s 2-tailed *t* test.) (**D**) Fibroblasts were transduced with control or Cre-expressing adenovirus for 72 hours and seeded with collagen gels to measure the percentage contraction of collagen gels after 6 hours (***P < 0.0005; *n* = 6; Student’s 2-tailed *t* test). (**E** and **F**) Fibroblasts were transduced with control or Cre-expressing adenovirus for 48 hours and treated with anti-Fas or control antibody for another 24 hours. The percentage of TUNEL-positive (red color) cells in the total cells (DAPI or blue color) was quantified using Elements image analysis software. Scale bar: 200 μm (****P < 0.00005, ***P < 0.0005; *n* = 3/group; 1-way ANOVA).

To examine whether Sox9 is required for the maintenance of fibroblast activation, we isolated activated fibroblasts from fibrotic lung lesions of Dox-treated *TGF*α*OE Sox9^fl/fl^* mice for 6 weeks. As shown in Figure 6A, we selectively deleted Sox9 in activated fibroblasts by treating them with Cre-expressing adenovirus and assessed the changes in ECM gene expression, contractility, and apoptosis. Western blot analysis indicated a significant loss in SOX9 protein levels in fibroblasts infected with Cre adenovirus compared with those in fibroblasts treated with control adenovirus for 72 hours (Figure 6B). *Sox9* knockdown resulted in a significant decrease in ECM-associated genes, including *Acta2*, *Col1*α*1*, and *Loxl2*, compared with those in control fibroblasts (Figure 6C). To measure the changes in the contractile function, we seeded fibroblasts infected with Cre or control adenovirus in collagen gels and observed a significant reduction in the contraction of collagen gels (Figure 6D). In addition, the TUNEL assays further confirmed that the knockdown of *Sox9* increased the number of TUNEL-positive cells in the presence of anti-Fas antibodies (Figure 6, E and F). Thus, our findings provide complementary evidence that Sox9 is required for the maintenance of the profibrotic functions of activated fibroblasts.

### Overexpression of Sox9 in myofibroblasts augments bleomycin-induced pulmonary fibrosis.

**Figure 7:**
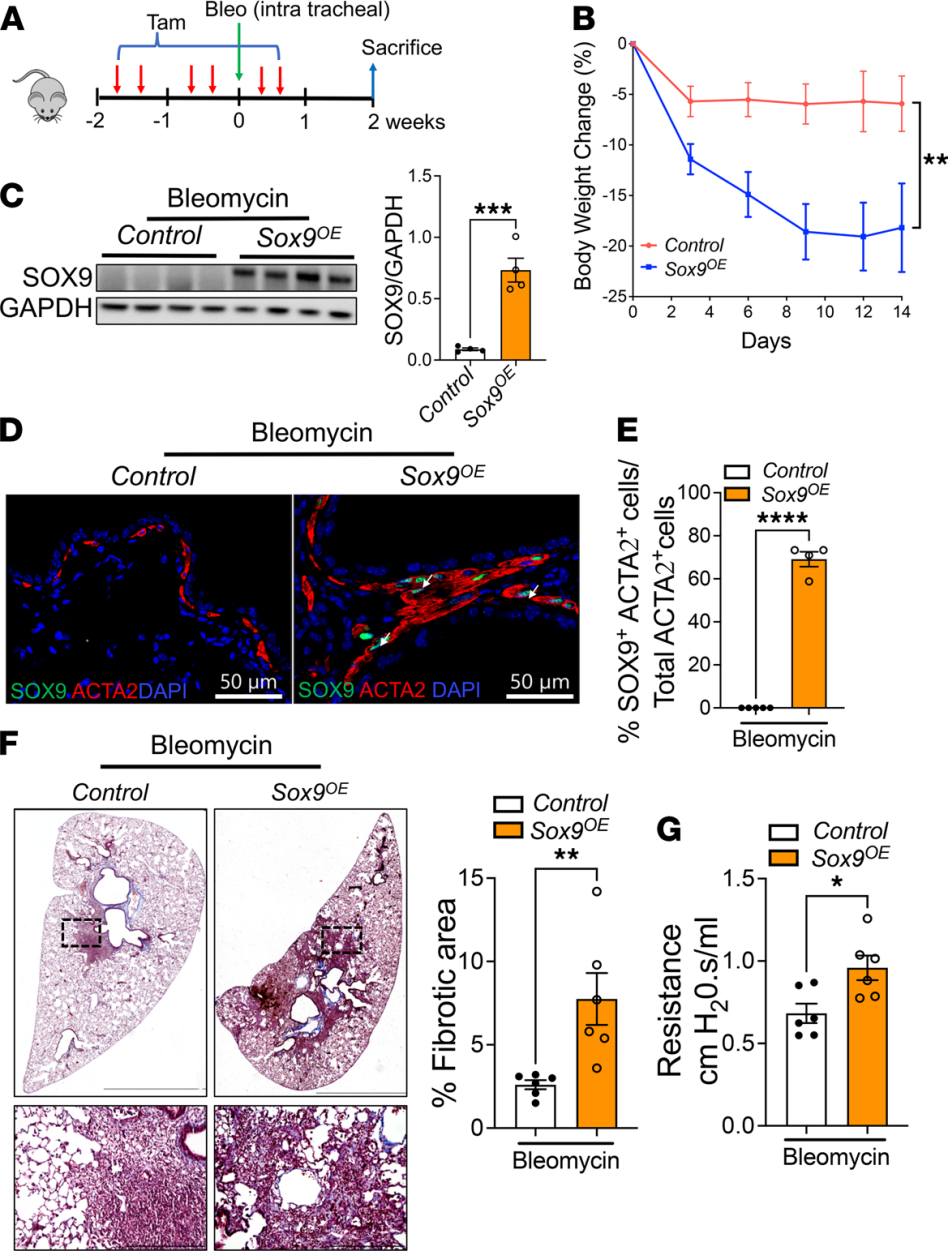
Overexpression of SOX9 in myofibroblasts augments bleomycin-induced pulmonary fibrosis. (**A**) Schematic representation of animal experiments with α*SMA^CreERT2^* (control) and α*SMA^CreERT2^ CAG-Sox9* (*Sox9^OE^*) mice treated with bleomycin and tamoxifen via intratracheal and intraperitoneal routes, respectively. (**B**) The progressive weight loss after bleomycin treatment in control and *Sox9^OE^* mice. (**P < 0.005; *n* = 6/group; 2-way ANOVA.) (**C**) The lung lysates of control and *Sox9^OE^* mice were immunoblotted with antibodies against SOX9 and GAPDH. SOX9 protein levels were normalized to GAPDH and are shown as fold induced change using a bar graph (***P < 0.005; *n* = 4; Student’s 2-tailed *t* test). (**D**) Representative confocal images of lung sections stained for SOX9 (green color), ACTA2 (red color), and DAPI (blue color). Scale bar: 50 μm. (**E**) The efficiency of SOX9 overexpression was quantified by counting the percentage of SOX9- and ACTA2-positive cells in total ACTA2-positive cells. (****P < 0.00005; *n* = 4; Student’s 2-tailed *t* test.) (**F**) Masson’s trichrome–stained lung sections of the control and *Sox9^OE^* mice treated with bleomycin. Images were captured at 4× original magnification and 20× original magnification with scale bars of 1500 and 200 μm, respectively. The percentage of the fibrotic area was quantified in the images of control and *Sox9^OE^* mice using BZ-X image analysis (**P < 0.005; *n* = 6; Student’s 2-tailed *t* test). (**G**) The lung resistance was measured using FlexiVent in control and *Sox9^OE^* mice treated with bleomycin (*P < 0.05; *n* = 6; Student’s 2-tailed *t* test).

**Figure 8:**
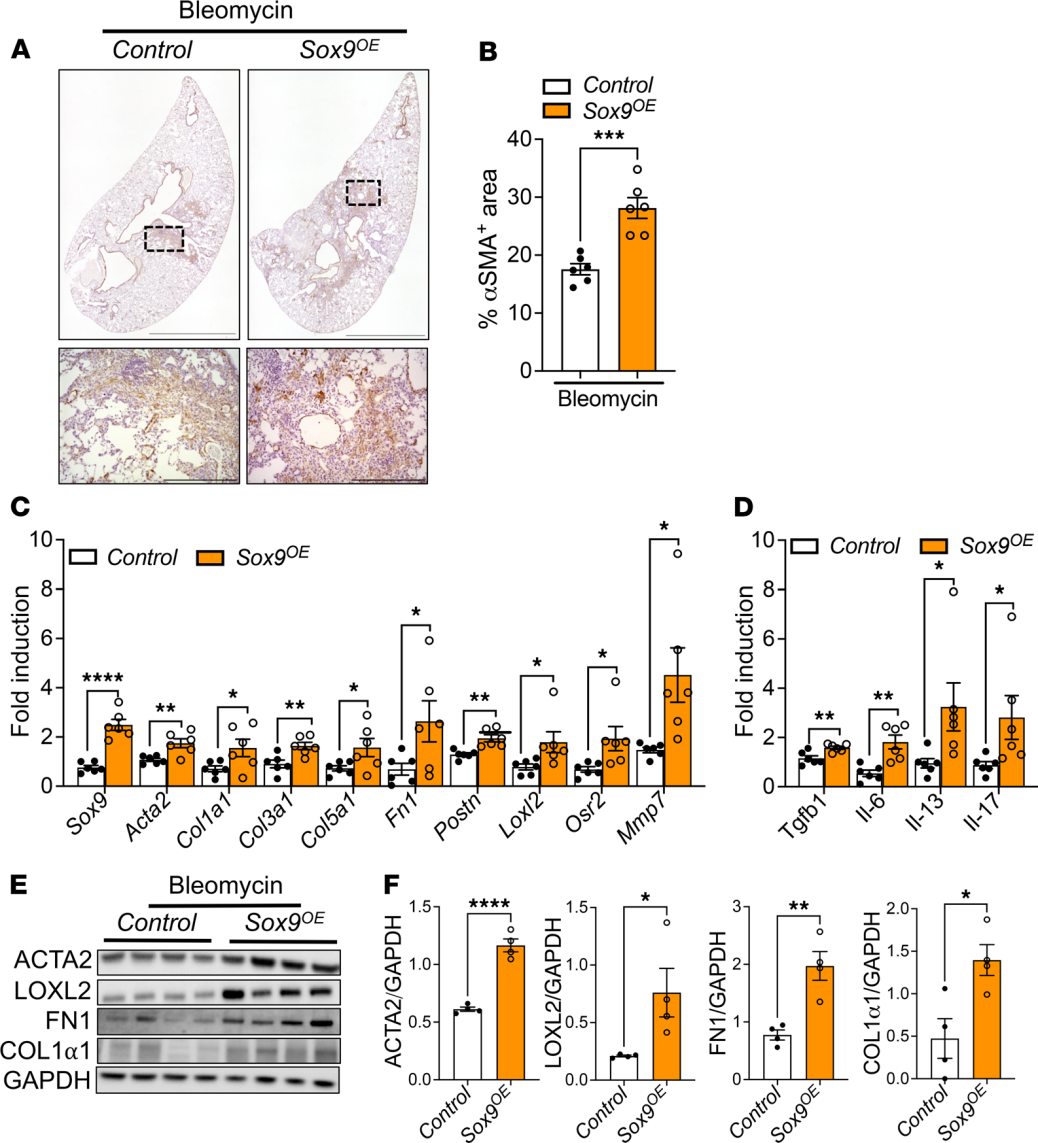
SOX9 overexpression in myofibroblasts augments bleomycin-induced pulmonary fibrosis in vivo. (**A**) Representative images of ACTA2-stained lung sections of *αSMA^CreERT2^* (control) and *αSMA^CreERT2^ CAG-Sox9* (*Sox9^OE^*) mice captured at 4× (low) and 20× (high) original magnification, respectively. Scale bars: 1500 and 200 μm. (**B**) Quantification of ACTA2-positive area in the whole lung section (***P < 0.0005, *n* = 6/group, Student’s 2-tailed *t* test). (**C** and **D**) Quantification of ECM and inflammatory-related gene transcripts among 2 groups. (****P < 0.00005, **P < 0.005, *P < 0.05, *n* = 6/group, Student’s 2-tailed *t* test.) (**E** and **F**) Immunoblots for ACTA2, LOXL2, FN1, COL1*α*1, and GAPDH expression in the whole lung tissue lysates of control and *Sox9^OE^* mice and quantification (****P < 0.00005, **P < 0.005, *P < 0.005, *n* = 4, Student’s 2-tailed *t* test). Immunoblots for Figure 7C and Figure 8E were from the same experiment, and blots were run in parallel to normalize with GAPDH levels.

To further substantiate our findings, we investigated whether Sox9 overexpression augments bleomycin-induced pulmonary fibrosis. We bred α*SMA^CreERT2^* mice with *CAG-Sox9* mice to generate myofibroblast-specific conditional Sox9-overexpressing mice (α*SMA^CreERT2^ Sox9^OE^*) and control mice (α*SMA^CreERT2^*). As shown in Figure 7A, the mice in both groups were treated with 2 tamoxifen injections per week for 2 weeks prior to intratracheal bleomycin instillation, and an additional injection was administered 1 week after bleomycin treatment. We monitored the changes in body weights after bleomycin instillation; the mice were sacrificed at the end of week 2 after bleomycin instillation. Notably, we observed a significant decline in the body weight of bleomycin-treated *Sox9^OE^* mice compared with bleomycin-treated control mice (Figure 7B). Levels of SOX9 protein were increased in the total lung lysates of bleomycin-treated *Sox9^OE^* mice compared with that in bleomycin-treated control mice (Figure 7C). To assess Sox9 upregulation in myofibroblasts, we coimmunostained lung sections with antibodies against SOX9 and ACTA2. We observed selective SOX9 overexpression in myofibroblasts of bleomycin-treated *Sox9^OE^* mice compared with that in myofibroblasts of bleomycin-treated control mice (Figure 7D). When we quantified the SOX9-positive myofibroblasts in total myofibroblasts that accumulated in the fibrotic lesions, we observed that a large percentage (~80%) of myofibroblasts overexpressed SOX9 in bleomycin-treated *Sox9^OE^* mice compared with bleomycin-treated control mice (Figure 7, D and E). Masson’s trichrome staining exhibited a substantial increase in collagen staining in the lung sections of bleomycin-treated *Sox9^OE^* mice compared with that in bleomycin-treated control mice (Figure 7F). We quantified the fibrotic area in the whole lung section from all mice and observed a significant increase in the percentage of the fibrotic area, normalized to the total scanned area in the lung sections of bleomycin-treated *Sox9^OE^* mice compared with that in bleomycin-treated control mice (Figure 7F). We measured the changes in lung resistance as a measure of lung function and observed significant deterioration of lung function of bleomycin-treated *Sox9^OE^* mice compared with that of bleomycin-treated control mice (Figure 7G). To assess myofibroblast accumulation, lung sections were immunostained with antibodies against ACTA2. ACTA2 staining increased in the lung parenchyma of bleomycin-treated *Sox9^OE^* mice compared with that in bleomycin-treated control mice (Figure 8A). We next quantified the ACTA2 staining area that was significantly increased in bleomycin-treated *Sox9^OE^* mice compared with bleomycin-treated control mice (Figure 8B). We also quantified the changes in ECM-associated genes in the total lung transcripts of bleomycin-treated *Sox9^OE^* and control mice. Consistent with changes in collagen levels, we observed a significant increase in transcripts of ECM-associated genes, including *Acta2*, *Col1a1*, *Col3a1*, *Col5a1*, *Fn1*, *Postn*, *Loxl2*, *Osr2*, and *Mmp7*, in bleomycin-treated *Sox9^OE^* mice compared with bleomycin-treated control mice (Figure 8C). We quantified the transcripts of several profibrotic growth factors, including *TGFb1*, *Il6*, *Il13*, and *Il17*, and found that their levels were elevated in the lungs of bleomycin-treated *Sox9^OE^* mice compared with those in the lungs of bleomycin-treated control mice (Figure 8D). Consistent with changes in transcript levels, we observed a marked increase in the levels of ACTA2, LOXL2, FN1, and COL1α1 in the lung lysates of bleomycin-treated *Sox9^OE^* mice compared with that in bleomycin-treated control mice (Figure 8, E and F). Taken together, our findings provide complementary in vivo evidence that Sox9 upregulation in fibroblasts is responsible for fibroblast activation and accumulation and plays an integral role in the pathogenesis of pulmonary fibrosis.

## Discussion

In this study, we showed that SOX9 was aberrantly activated in fibroblasts of distal lung fibrotic lesions in both human IPF and a mouse model of TGF-α–induced pulmonary fibrosis. We demonstrated that the TGF-α/WT1 axis was involved in SOX9 upregulation in pulmonary fibrosis. Using in vitro fibroblast activation assays, we showed that SOX9 upregulation caused excessive migration, FMT, ECM gene expression, and resistance to apoptosis in IPF fibroblasts. To investigate the in vivo relevance of Sox9 upregulation in fibroblasts, we performed fibroblast- or myofibroblast-specific deletion or overexpression of Sox9. Both loss-of-function and gain-of-function studies suggest a pathological role for Sox9 in fibroblast activation and pulmonary fibrosis in 2 alternative mouse models of pulmonary fibrosis.

Recent advances in quantitative transcriptomics using next-generation RNA-Seq and single-cell genomics have helped us identify the loss of lineage specificity in transcription factors between proximal and distal lung cells in IPF pathogenesis (12, 13). Particularly, fibroblast dysregulation is important in IPF due to its direct involvement in excessive collagen deposition and the progressive expansion of fibrotic lung lesions in IPF (14, 15). Here, we report a pathogenic fibroblast population that overexpressed SOX9, a key transcription factor that is thought to be epithelial progenitor cell specific during lung development or in adult lungs. We showed that SOX9-positive fibroblasts and myofibroblasts accumulated in the distal areas of IPF lungs, including fibroblastic foci and subpleural fibrotic lesions. Consistent with the findings in IPF lungs, we found that these SOX9-expressing fibroblasts accumulated in the distal fibrotic lung lesions during TGF-α–induced pulmonary fibrosis. However, in normal lung sections, we observed limited or no expression of SOX9 in fibroblasts or myofibroblasts. Mechanistically, using the *SOX9* promoter-driven luciferase assays, we showed that the TGF-α/WT1 axis was responsible for the upregulation of Sox9 in fibroblasts, which resulted in an increase in migration, FMT, survival, and ECM gene expression. We demonstrated that both bleomycin- and TGF-α–induced pulmonary fibrosis depend on SOX9-driven increases in profibrotic gene networks and fibroblast activation. Together, these studies identified SOX9 as a positive regulator of fibroblast activation in the pathogenesis of pulmonary fibrosis, illustrating the potential utility of targeting SOX9 or SOX9 downstream targets in the treatment of IPF and other fibrotic diseases.

The Sox family of transcription factors in airway epithelial cells, including SOX2 and SOX9, has been extensively studied in the context of proximal-distal patterning of the lung (16, 17). Specifically, Sox9 plays a critical role in lung branching morphogenesis and is highly expressed during the pseudo glandular stage in epithelial progenitor cells. Later, its expression is downregulated with the onset of alveolarization, and limited or no expression of SOX9 is observed in either epithelial cells or mesenchymal cells of adult distal lungs (6, 9). Our findings demonstrate that SOX9 is upregulated in the distal lung fibroblasts of IPF. Importantly, our findings showed that multiple profibrotic growth factors, including *BMP2*, *CTGF*, and *TGF*α, can upregulate *SOX9*. Previous studies identified the role of BMPs and FGFs in upregulating SOX9 in epithelial progenitor cells during lung development, whereas the role of TGF-α remained unclear (18, 19). During TGF-α–induced pulmonary fibrosis, WT1-positive fibroblasts have been shown to accumulate in distal fibrotic lesions, similar to SOX9-positive fibroblasts (3, 4). Recent studies from our lab and others showed that WT1 upregulation in fibroblasts is involved in fibroblast activation and the pathogenesis of pulmonary fibrosis (3, 4, 20). Here, we demonstrate that the loss of WT1 is sufficient to attenuate TGF-α–induced SOX9 expression in fibroblasts. Additionally, we performed bioinformatics analysis on the *SOX9* promoter region and identified a potential binding site for WT1. Using luciferase reporter assays, we demonstrated that WT1 indeed binds to the *SOX9* promoter and functions as a positive regulator of SOX9 expression in fibroblasts. However, it is important to note that multiple growth factors can upregulate SOX9 in fibroblasts and epithelial cells (17, 21). Therefore, we cannot rule out the possibility that other mechanisms are involved in SOX9-induced pulmonary fibrosis. Although we indicated a role for SOX9 in fibroblast activation and pulmonary fibrosis, further work is necessary to determine the impact of SOX9 in other lung cells, including epithelial cells, during the initiation, maintenance, and resolution of pulmonary fibrosis.

IPF is a severe fibrotic disease in which fibroblasts are transformed into an activated state, called myofibroblasts, and deposit excess ECM in response to chronic injury; this process ultimately leads to the formation of scar tissue (22–24). Activated fibroblasts are highly proliferative, secrete excessive ECM, are invasive and migrative, and are resistant to apoptosis (25). Myofibroblasts are the main culprits involved in the progression of fibrosis. Lineage-tracing studies on the origins of myofibroblasts have shown that tissue-resident fibroblasts, including WT1-positive fibroblasts, are a source of myofibroblasts (4, 26). However, SOX9-driven gene networks in fibroblasts that cause the pathological features observed in IPF are poorly defined. Therefore, we performed transcriptional profiling of SOX9 knockdown in IPF fibroblasts and identified SOX9-driven gene networks and biological processes that are dysregulated in IPF fibroblasts. Particularly, enrichment analysis of SOX9-knockdown-downregulated genes with IPF gene data sets indicated the role of SOX9 in ECM production and organization, migration, FMT, and survival of the fibroblasts. In support of its profibrotic actions, SOX9 knockdown in IPF fibroblasts resulted in reduced migration and downregulation of several ECM-related genes. Similar observations were made in mouse fibrotic fibroblasts, where SOX9 affected ECM gene expression. Notably, SOX9 regulates the expression of LOXL2, an important ECM-organizing enzyme that plays a crucial role in matrix remodeling and fibrogenesis (27). Previous studies have demonstrated that LOXL2 is a marker for IPF progression, and its inhibition resulted in the slower progression of the disease (27–29). SOX9 is well recognized as a transcriptional regulator for various ECM-related genes based on studies related to chondrogenesis and cancer cell types (30, 31). We have highlighted the role of SOX9 in FMT and migration of distal fibroblasts by regulating the expression of DPYSL3 and mitogens, such as FGF1, whose levels are highly increased in IPF fibroblasts (32). Using fibroblasts with gain or loss of SOX9 expression, we showed that SOX9 functions as a positive regulator of ACTA2 expression and regulates the contractility of collagen gels. Likewise, in the FMT assay, SOX9 overexpression promoted FMT. These findings suggest that the profibrotic activity of SOX9 may, at least in part, be attributed to the induction of ACTA2. However, the mechanisms underlying SOX9-driven ACTA2 expression and contractility remain to be investigated in fibroblasts. Previous studies have identified increased expression of the BCL family of antiapoptotic genes, including BCL-2, BCL-XL, and BCL2L2, in IPF fibroblasts (33). These genes contribute to resistance against Fas-mediated apoptotic clearance in fibroblasts and myofibroblasts (34–37). Therefore, we analyzed the role of SOX9 in fibroblast survival; our findings suggest that SOX9 promotes the survival of fibroblasts by inducing the expression of antiapoptotic genes (*BCL-XL* and *BCL-2L2*). Strikingly, the resistance against Fas-induced apoptosis observed in IPF fibroblasts was reversed with the loss of SOX9. Thus, our results indicate that enhanced expression of SOX9 in IPF and TGF-α fibroblasts allows the cells to resist Fas-mediated apoptosis. Consequently, survival and accumulation of activated fibroblasts likely occur in IPF during TGF-α–induced pulmonary fibrosis. Overall, our findings indicate that SOX9 is a positive regulator of fibroblast activation by inducing migration, FMT, survival, and production of ECM in pulmonary fibrosis.

Previous studies have identified the important roles of WT1 in both TGF-α– and bleomycin-induced pulmonary fibrosis, whereas the role of SOX9 has remained unclear. To determine whether SOX9 alters TGF-α– and bleomycin-induced pulmonary fibrosis, we generated conditional fibroblast- or myofibroblast-specific *Sox9*-knockout or -overexpressing mice. We employed a commonly used *Col1*α*2^CreERT^* fibroblast-specific Cre driver to delete Sox9 in fibroblasts (38, 39). Strikingly, the increase in TGF-α–induced fibroblast activation and pulmonary fibrosis in *TGF*α*OE* mice was attenuated with the loss of SOX9, which supports the profibrotic function of SOX9. Consistent with our findings, few studies have demonstrated that either ECM proteins or loss of SOX9 affects the cytoskeletal organization and interferes with cell morphology (6, 40, 41). In addition, we documented the important role of SOX9 in fibroblast activation using an alternative mouse model of bleomycin-induced pulmonary fibrosis. In IPF, myofibroblasts are the ultimate differentiated cells that deposit excess ECM components and destroy the architecture of the lung; therefore, we overexpressed SOX9 in myofibroblasts (4, 42). Our potentially novel findings demonstrated that SOX9 overexpression led to a decrease in body weight compared with control mice and a decline in lung function. Additionally, the histology of the lung sections indicated an increase in collagen deposition with an increase in SOX9 levels; a parallel increase in the ECM-related proteins accompanied the escalation of fibrosis. Interestingly, SOX9 overexpression in myofibroblasts led to an increase in the profibrotic factors such as TGF-β1 and inflammatory cytokines (*Il6*, *Il-13*, and *Il-17*), resulting in tissue remodeling. TGF-β1 is considered a master regulator of fibrosis and is secreted by injured epithelial and inflammatory cells and partly by (myo)fibroblasts in patients with IPF (43–45). Numerous studies have shown a positive association between TGF-β1 and Sox9 expression or stabilization (46, 47). Our studies suggest that SOX9 regulates the expression of these profibrotic factors in the myofibroblasts either directly or indirectly and likely forms a positive feed-forward loop, resulting in progressive fibrosis. A recent study on IPF fibroblasts confirmed that fibroblasts also secrete Il6 and activate STAT3/SMAD3 signaling, which could trigger additional fibrotic events (48). However, the mechanisms by which SOX9 regulates these factors have yet to be determined. Additionally, the role of SOX9 in the epithelial progenitor population in IPF must be determined to therapeutically target this molecule. The pathogenic role of SOX9 in inducing fibrosis is confirmed in other organs, such as the liver, kidney, and heart (31, 49–51). In the heart, SOX9 is predominantly expressed by cardiomyocytes and cardiac fibroblasts after myocardial infarction injury in mice. Importantly, fibroblast-specific SOX9 deletion was sufficient to attenuate migration, proliferation, and contractility of cardiac fibroblasts and improve cardiac function (51). Likewise, we found that the loss of SOX9 attenuated migration and contractility but not the proliferation in lung fibroblasts (our unpublished data), further suggesting the importance of SOX9-positive fibroblasts as a part of the profibrotic response in multiple organs. In the liver, the deletion of SOX9 in vivo has been shown to attenuate CCL4-induced liver fibrosis (49). Importantly, elevated levels of SOX9 or SOX9-regulated matrix proteins have been shown to correlate with the progression toward cirrhosis in these patients (31, 49). Our genomic studies have identified several downstream targets of SOX9 that may have an important role in predicting the disease progression or mediating the pathology in lung fibrosis. To substantiate our findings, we further evaluated upregulation of SOX9 and its targets in an independent cohort data set of 160 IPF patients and 108 controls (GSE47460) as described previously (52). Consistent with our findings, we observed a significant increase in *SOX9* transcripts levels in the lungs of IPF compared with healthy controls (Supplemental Figure 6A). Several of the SOX9-dependent ECM genes, including *COL15A1*, *COL14A1*, *SFRP2*, *OSR2*, *ASPN*, *COL3A1*, *ACTA2*, *OGN*, and *LOXL2*, were found to be upregulated and positively correlated with Sox9 levels (Supplemental Figure 6B). Further, the levels of Sox9 and its target genes were negatively correlated with the forced vital capacity and the diffusing capacity of the lung for carbon monoxide (Supplemental Figure 6C). However, further studies are needed to identify the potential associations between cell-specific SOX9 targets and the decline in lung function, which could potentially assist in the stratification of patients with IPF. Nevertheless, the pathological role of SOX9 in activating fibroblasts and orchestrating fibrosis in multiple organs might open up opportunities for drug discovery, precision targets, and therapeutic interventions (31, 49–51).

Taken together, our results established multiple roles for SOX9 in fibroblasts that promote fibrosis. We demonstrated that SOX9 is a positive regulator of migration, ECM production, and organization, which promotes FMT processes and allows fibroblasts to resist apoptosis. Importantly, using 2 complementary mouse models of pulmonary fibrosis, we demonstrated that SOX9 upregulation in the distal lung fibroblasts causes severe fibrotic lung disease. These new findings highlight how excessive SOX9 activity in fibroblasts can contribute to the progressive expansion of fibrotic lesions in the lung and that targeting the aberrant activation of SOX9 could be a therapeutic strategy to mitigate ongoing pulmonary fibrosis in IPF.

## Methods

### Mouse models of TGFα^OE^ and bleomycin-induced pulmonary fibrosis.

Floxed *Sox9*, *WT1* mice, and *Col1*α*2^CreER^* transgenic mice were characterized previously (53–56). The *WT1*-flox mice were a gift from Christoph Englert from Molecular Genetics lab, Leibniz Institute, Jena, Germany. To generate fibroblast-specific Sox9-deficient mice, *Sox9^fl/fl^* mice were bred with heterozygous *Col1*α*2^CreER^* mice to generate *Col1*α*2^CreER^* *Sox9^fl/fl^* mice. The generation of Dox-inducible and Clara-cell-specific TGF-α–overexpression mice (herein *TGF*α*OE* ) was previously described (57). We bred *TGF*α*OE* mice with *Col1*α*2^CreER^ Sox9^fl/fl^* mice to generate *CCSP-rtTASox9^fl/fl^* (control mice), *TGF*α*OE Sox9^fl/fl^*, and *TGF*α*OE Col1*α*2^CreER^ Sox9^fl/fl^* mice. All the mice used were aged between 11 and 16 weeks in our animal studies. Mice were fed a diet containing Dox (62.5 mg/kg) to induce TGF-α overexpression, which causes the mice to develop a significant amount of fibrotic lung disease by 4 to 6 weeks. To induce cell-specific CreER-driven recombination, mice were administered 100 μL of tamoxifen (100 mg/kg) twice weekly via i.p. injection, as described previously (4). The generation of *Sox9^OE^* and α*SMA^CreERT2^* mice has been described previously (58, 59). We bred *Sox9^OE^* mice with α*SMA^CreERT2^* mice to generate α*SMA^CreERT2^* *Sox9^OE^* and α*SMA^CreERT2^* (control) mice. As mentioned previously, tamoxifen was administered via the i.p. route twice in 1 week before 2 weeks of intratracheal bleomycin (3 U/kg) instillation and for a week after bleomycin injury; the mice were sacrificed after 2 weeks of bleomycin instillation. To generate *Col1*α*2^CreER^* *WT1^fl/fl^* mice, we bred *Col1*α*2^CreER^* mice with *WT1^fl/fl^* and generated lung fibroblasts by culturing lungs in vitro as detailed in our published studies (60).

Lung function parameters were measured using Flexi Vent (SCIREQ Inc.). The mice were sacrificed, and the lungs were collected for histology, RNA, protein, and hydroxyproline. Hydroxyproline from the right lung was measured using colorimetry, as described previously (61).

### Human samples.

IPF and nonfibrotic lung specimens were obtained from the Interstitial Lung Disease Biorepository at the University of Michigan Medical School and provided by Steven Huang in the Division of Pulmonary and Critical Care Medicine. IPF was diagnosed according to the guidelines of the American Thoracic Society (62) and confirmed in a multidisciplinary fashion through weekly consensus conference. All tissues were acquired using research protocols that were approved by the Michigan Medicine Institutional Review Board (HUM00105694) and all patients provided informed consent. Lung tissue was obtained from explanted lungs after lung transplantation.

### RNA isolation and real-time PCR.

The RNA from tissues and primary cells was isolated using an RNeasy kit (QIAGEN) as per the instructions described in a previous study (63). cDNA was synthesized, and real-time PCR was performed using the SYBR select master mix (Bio-Rad) and CFX384 Touch Real-Time PCR instrument (Bio-Rad) and analyzed using CFX maestro software version 4.0. The target gene transcripts from mice and humans were normalized to either hypoxanthine-guanine phosphor ribosyl transferase or human β-actin, respectively. The primer lists for both human and mouse genes are provided in Supplemental Tables 2 and 3, respectively.

### Histology and immunohistochemistry.

Histological procedures were performed as mentioned previously (4). Briefly, paraffin-embedded tissues were cut into 5 μm sections and deparaffinized for Masson’s trichrome staining. For immunohistochemistry, lung sections were deparaffinized followed by citric acid antigen retrieval (pH 6.0) and methanol treatment, blocked in 5% donkey serum, incubated with the primary antibody overnight, incubated with species-specific secondary peroxidase antibodies, and then stained with DAB. Bright-field images were captured through Keyence BZ series microscopy and quantified using a BZ-X analyzer.

### Immunofluorescence and confocal imaging.

Isolated lungs or tissues were fixed overnight with 4% paraformaldehyde at 4°C, followed by PBS washes. Thereafter, the tissues were cryoprotected with 30% sucrose and embedded in OCT (Tissue-Tek). The procedure for immunofluorescence was performed as previously described (10). Briefly, we performed citric acid antigen retrieval (pH 6.0) and blocked the sections using 5% donkey serum, followed by overnight incubation of the samples with specific primary antibodies. The next day, samples were incubated with species-specific donkey Alexa Fluor 488, 568, or 647 antibodies, and DAPI was applied for nuclear staining. Primary cells were fixed with 4% paraformaldehyde for 10 minutes. Images were captured using a Nikon AIR-A1 confocal microscope and analyzed using the Imaris or Elements software. Details of antibodies used and their dilutions are provided in Supplemental Table 4.

### TUNEL staining.

Depending on the experiment, human or mouse fibrotic fibroblasts were treated with siRNA or transduced with adenovirus, respectively, for 48 hours. Cells were then treated with species-specific anti-Fas antibody (250 ng/mL, 05-201, clone CH11, or MilliporeSigma) for 24 hours. Nuclei were stained using DAPI, and DNA fragmentation of apoptotic cells was performed using the terminal deoxynucleotidyl TUNEL method using an In Situ Cell Death Detection kit, TMR red (12156792910, Roche Diagnostics); staining was performed according to the manufacturer’s instructions. The images were captured at 20× original magnification using a confocal microscope and quantified using Elements software version 9.1.

### Western blot.

Western blotting analysis was performed as described previously (61) using cell lysis buffer (Cell Signaling Technology). Briefly, the lysates were electrophoresed and then transferred onto a nitrocellulose membrane. The membranes were blocked and incubated with primary antibodies overnight, which are listed with their dilutions in Supplemental Table 3. The Western blots were visualized and quantified using phosphorimager software, Multi gage (Fujifilm). The target proteins were normalized with GAPDH.

### Primary fibroblast cultures and treatments.

Primary fibroblasts from lung tissues of humans and mice were isolated using collagenase digestion, as described previously (4), then cultured in DMEM (10% FBS) for human fibroblasts or IMDM for mouse fibroblasts (5% FBS). The fibroblasts used in the experiments were between passages 1 and 4. Cells were treated with TGF-α (100 ng/mL, 100-16A, PeproTech), BMP2 (200 ng/mL, 355-BM-010, R&D Systems), IGF-1 (100 ng/mL, 291-G1-200 R&D Systems), or CTGF (50 ng/mL, SRP4702, MilliporeSigma) overnight at low-serum conditions. EGFR pathway inhibitors were used as described in our published studies with few modifications (64). Briefly, fibroblasts were pretreated with 0.1 μM ARRY (MEK inhibitor) or PX-866 (PI3K inhibitor) before 1 hour of stimulation with TGF-α and the appropriate controls for different periods.

### Transfection and transduction of cells.

For stealth siRNA-mediated studies, IPF fibroblasts were transfected with stealth siRNA control (catalog 12935300) or target siRNA (Sox9: HSS110100, WT1: HSS111390, Invitrogen) for 72 hours using a Lipofectamine 3000 transfection kit (Life Technologies) as described previously (10). Similarly, using Lipofectamine 3000, we transfected the plasmids for the luciferase assays. Depending on the experiment, human primary fibroblasts were treated with a control virus, Sox9, or WT1-overexpressing viral particles. Cells were harvested after 48 to 72 hours for RNA or protein lysates, as mentioned previously (10). For Cre-mediated in vitro experiments, *TGF*α*OE* and *Sox9^fl/fl^* mice were treated with Dox for 6 weeks, and the lung fibroblasts were cultured. The purified fibroblasts were transduced with either adeno-Cre-expressing adenovirus or control adeno viral particles to excise *loxP* sites that flanked the *Sox9* gene for 48 to 72 hours. Then, we performed the gel contraction assay, TUNEL assay, and RNA isolation.

### Migration assay.

The IPF lung fibroblasts were transfected with either siControl or si*SOX9* siRNA for 48 hours, and a scratch was made in the wells. Migration was measured using the IncuCyte Zoom real-time imaging system for the next 48 hours. Images were acquired every 3 hours on 10× original magnification, and the percentage of wound closure was measured using IncuCyte Zoom software 2018A.

### Myofibroblast differentiation assay.

Primary lung fibroblasts were isolated from α*SMA^CreERT2^* and *Rosa^mTmG^* (65) for the fibroblast-to-myofibroblast transformation assay, as described previously (60). Briefly, the fibroblasts were transduced with either control lentivirus or Sox9-overexpressing lentiviral particles and were treated with 4-hydroxy-tamoxifen (2 μM, H7904, MilliporeSigma) for 72 hours. Then, cells were fixed with 4% paraformaldehyde, and the nucleus was stained with DAPI. Confocal images were obtained at 20× original magnification, and, for quantification of myofibroblasts, the MetaMorph imaging software (Molecular Devices) was employed.

### Collagen contraction assay.

Depending on the experiment, after 48 hours of transfection or infection, fibroblasts were seeded into rat tail collagen (1 mg/mL, A10483-01, Gibco) gel matrices, as described previously (66). Briefly, gels were detached from the wells, images were captured using a stereomicroscope, and the area of contraction was measured using ImageJ software.

### Luciferase activity assay.

Luciferase assay was performed as previously described (10). Briefly, HEK293 cells (American Type Culture Collection) were transfected with an empty promoter vector (S790005, Switchgear Genomics), human *Sox9* promoter (*SOX9^WT1^*) (S705795, Switchgear Genomics), or WT1-binding element deleted *Sox9* promoter (*SOX9*^Δ^*WT1*) with Renilla luciferase reporter for 48 hours. Then, the cells were adapted to low-serum DMEM-F12 media followed by treatment with TGF-α for 24 hours. *SOX9*^Δ^*WT1* that lacked 16 bp (–327 to –311) from *SOX9^WT1^* was generated using a site-directed mutagenesis kit (catalog 200524, QuikChange II Site-Directed Mutagenesis Kit, Agilent Technologies). In another experiment, the cells were cotransfected with either pLJM1 control or pLJM1 WT1-overexpressing lentivector with an empty promoter, *SOX9* promoter, or *SOX9*^Δ^*WT1* promoter for 48 hours and with TGF-α for 24 hours with appropriate controls. The luciferase assay was performed using a LightSwitch Luciferase Assay kit (Switchgear Genomics) and a Glomax luminometer (Promega).

### RNA-Seq and bioinformatic analysis.

RNA-Seq was performed using RNA isolated from the Sox9-knockdown studies in IPF fibroblasts. The RNA-Seq method and differential gene expression analysis have been described previously (4). The RNA-Seq data were deposited in the National Center for Biotechnology Information Gene Expression Omnibus with the ID GSE171532. Briefly, Sox9 siRNA-generated gene signatures were intersected with available IPF gene data sets (GSE53845; ref. 11). Enrichment analysis was performed on downregulated genes with Sox9 siRNA and upregulated in IPF using ToppFun application of the ToppGene Suite (67).

### Statistics.

For multiple comparisons, 1-way ANOVA with post hoc Tukey’s test or 2-way ANOVA with post hoc Sidak’s test was performed. Student’s 2-tailed *t* test was used to determine statistical significance among 2 groups. *P* values of less than 0.05 were considered statistically significant. The averaged or mean data are presented with SEM to indicate variability. For SOX9 and its target gene correlation studies, we used Pearson coefficient analysis.

### Study approval.

All animal experiments were performed under protocols approved by the Institutional Animal Care and Use Committee of Cincinnati Children’s Hospital Research Foundation, Cincinnati, Ohio, USA. Informed consent was obtained from the patients undergoing lung transplants, and the study protocols were approved by the Michigan Medicine Institutional Review Board, Ann Arbor, Michigan, USA (HUM00105694).

## Author contributions

PRG and SKM devised the project, were involved in designing and executing the experiments, analyzed data, and wrote the manuscript; PRG performed most of the experiments; D Soundararajan performed immunostaining and cloning experiments; AGJ performed bioinformatic analysis and edited the manuscript; SKH provided human lung tissues and edited the manuscript; D Sinner shared the Sox9^OE^ mice and edited the manuscript; and RKK performed real-time PCR and immunostaining experiments.

## Supplementary Material

Supplemental data

## Version 1 09/14/2021

In-Press Preview

## Version 2 10/22/2021

Electronic publication
