# Supplementary material for: Dysregulated overexpression of Sox9 induces fibroblast activation in pulmonary fibrosis
Source: JCI Insight. 2021 Oct 22;6(20):e152503. doi: 10.1172/jci.insight.152503 (PMC8564901; doi:10.1172/jci.insight.152503)
Supplement: Supplemental data [file jciinsight-6-152503-s148.pdf]

# Dysregulated overexpression of Sox9 induces fibroblast activation in pulmonary fibrosis

Prathibha R. Gajjala[1,2], Rajesh K. Kasam[1,2], Divya Soundararajan[1,2], Debora Sinner[1,3], Steven K. Huang[4], Anil G. Jegga[1,5] and Satish K. Madala[1,2]*

[1] Department of Pediatrics, University of Cincinnati, College of Medicine, Cincinnati, OH, USA

[2] Division of Pulmonary Medicine, Cincinnati Children's Hospital Medical Center, Cincinnati, OH, USA

[3] Division of Neonatology and Pulmonary Biology, Perinatal Institute, Cincinnati Children's Hospital Medical Center, Cincinnati, OH, USA

[4] Division of Pulmonary and Critical Care Medicine, University of Michigan Medical School, Ann Arbor, MI, USA

[5] Division of Biomedical Informatics, Cincinnati Children's Hospital Medical Center, Cincinnati, OH, USA

*** Correspondence:** Satish K. Madala, Division of Pulmonary Medicine, Cincinnati Children's Hospital Medical Center, MLC 2021, 3333 Burnet Avenue, Cincinnati, OH 45229. Email: satish.madala@cchmc.org Phone: (513) 636-9852 Fax: (513) 636-9946

## Supplemental data

Table of contents:
Supplemental Figures 1-6 (Page 2-7)
Supplemental Table S1-4 (Page 8-14)

**Supplemental Figure 1**

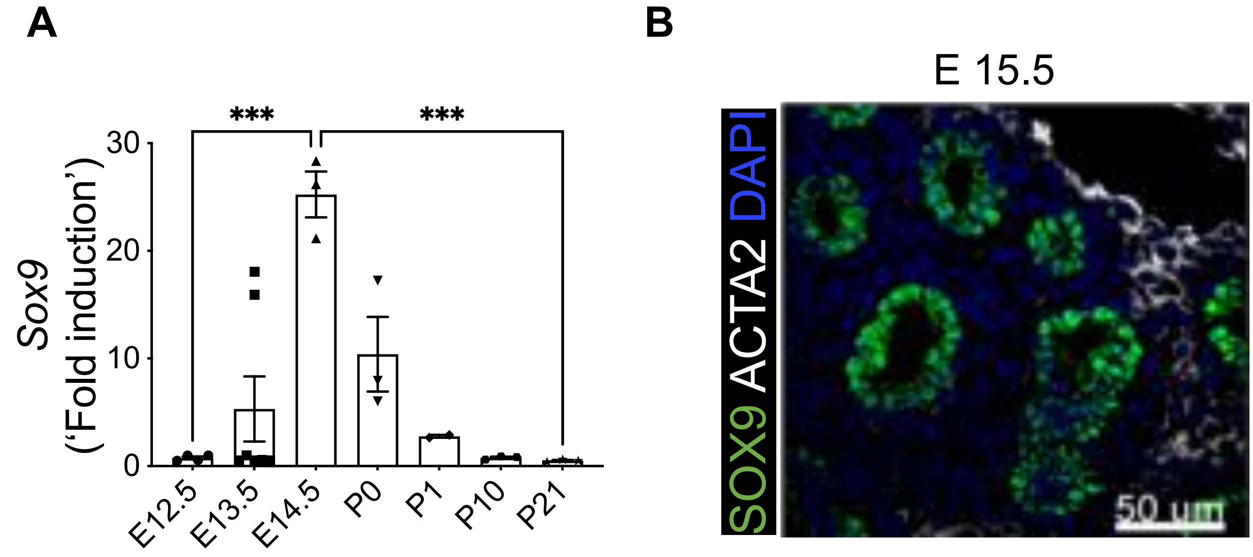


**Supplemental Figure 1. SOX9 is selectively upregulated in the distal lung epithelial progenitor cells.** (A) Quantification of *Sox9* transcripts at different stages of lung development by RTPCR. (***P <0.0005, n = 2-7/group, unpaired t-test). (B) Lung sections of $\alpha SMA^{YFP}$ mice embryos at E15.5 stained for SOX9 (green) and ACTA2 (white). Scale bar: 50 μm.

**Supplemental Figure 2**

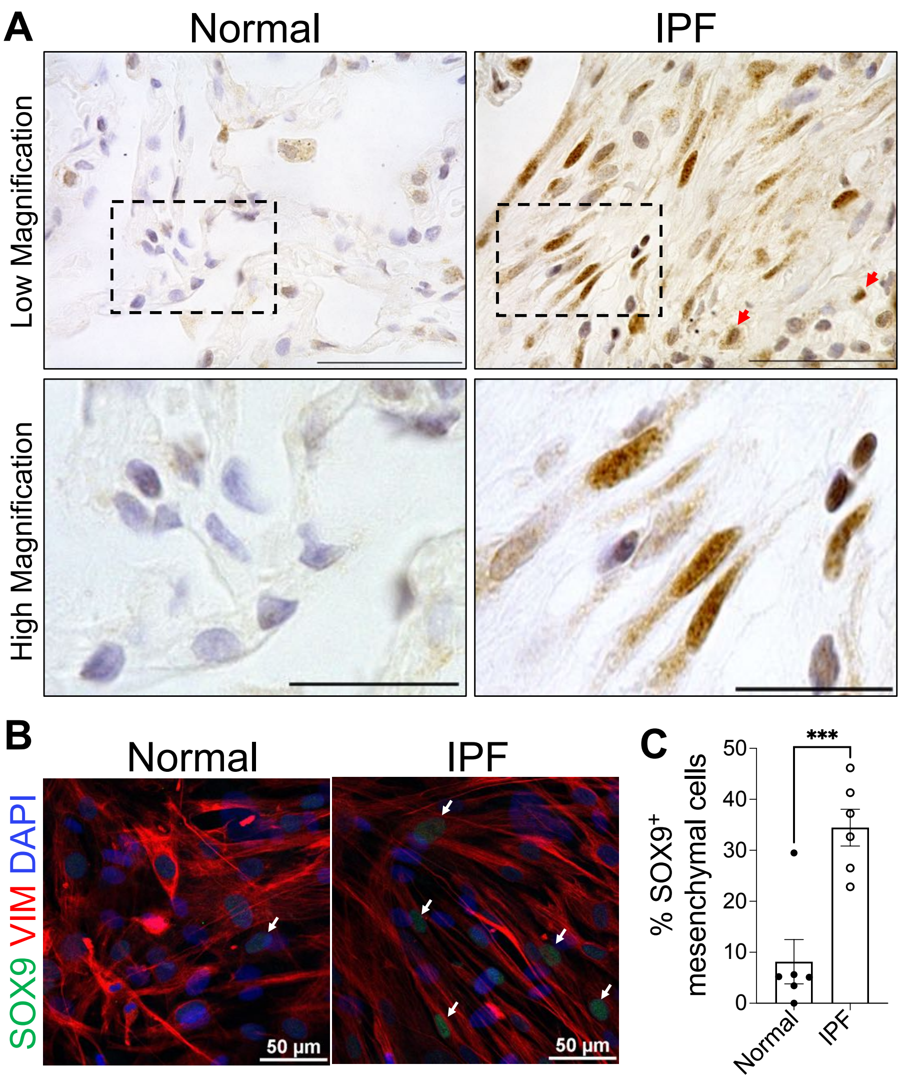


**Supplemental Figure 2. SOX9 upregulation in the distal lung cells of IPF.** (A) Representative immunostaining images of SOX9 in the lung sections of normal and IPF subjects obtained at 100x magnification. Scale bar: 50µm and 20 µm. Red arrows indicate the non-spindle shaped cells positive for SOX9. (B) Co-immunostaining of SOX9 and vimentin in the distal lung fibroblasts from IPF and normal lung cultures indicated with white arrows. (C) Quantification of SOX9 (green) and vimentin (red) dual positive cells in total fibroblasts from IPF and normal lung cultures (***P <0.0005, n = 6, unpaired t-test).

## Supplemental Figure 3

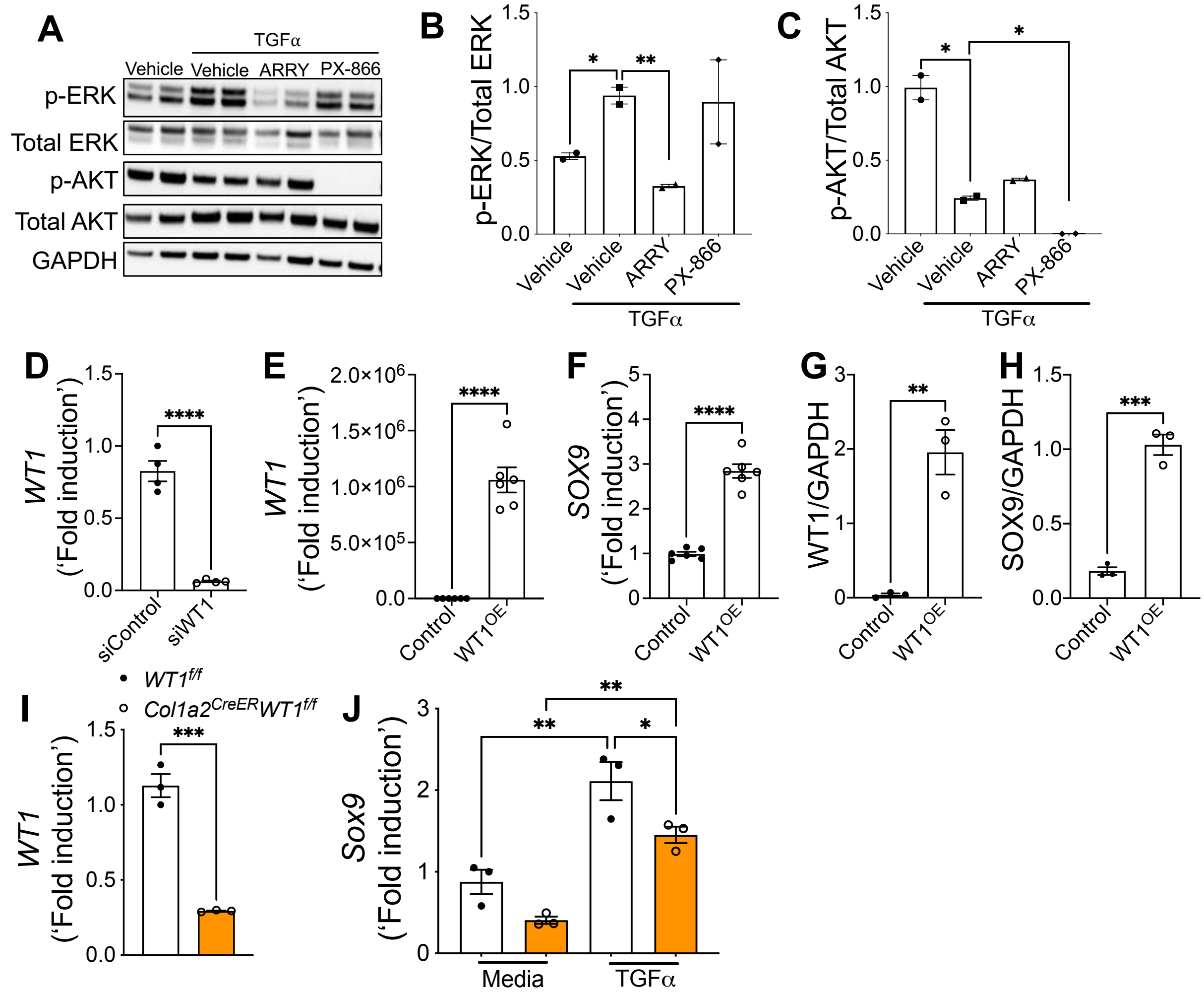


**Supplemental Figure 3. SOX9 is upregulated by TGFα-driven signaling and the transcription factor WT1.** (A-C) Western blot analysis for pERK44/42, pAKT, ERK44/42, AKT and GAPDH in the lysates of IPF fibroblasts treated with vehicle, ARRY (MEK inhibitor) and PX-866 (PI3K inhibitor) in presence or absence of TGFα for 30 minutes (**P <0.005, *P <0.05; n = 2; one-way ANOVA). (D) Quantification of *WT1* transcripts in IPF fibroblasts treated with either control or *WT1* specific- siRNA for 72 hrs. (****P <0.00005; n = 4; unpaired t-test). (E and F) Quantification of *WT1* and *SOX9* transcripts in the normal lung fibroblasts transduced with control or WT1 over expressing adeno virus for 72 hrs. (****P <0.00005; n = 4; unpaired t-test) (G and H) Quantification of WT1 and SOX9 protein levels in the lysates of normal lung fibroblasts transduced with control or WT1 over expressing adeno virus for 72 hrs. (***P <0.0005, **P <0.005 n = 3, unpaired t-test. (I and J) Quantification of *WT1* and *Sox9* transcripts in the normal mouse lung fibroblasts of $WT1^{f/f}$, and $Col1\alpha2^{CreER}WT1^{f/f}$ mice treated with 4-hydroxy tamoxifen for 72 hours and treated with TGFα overnight (**P <0.005, *P <0.05; n = 3; one-way ANOVA).

**Supplemental Figure 4**

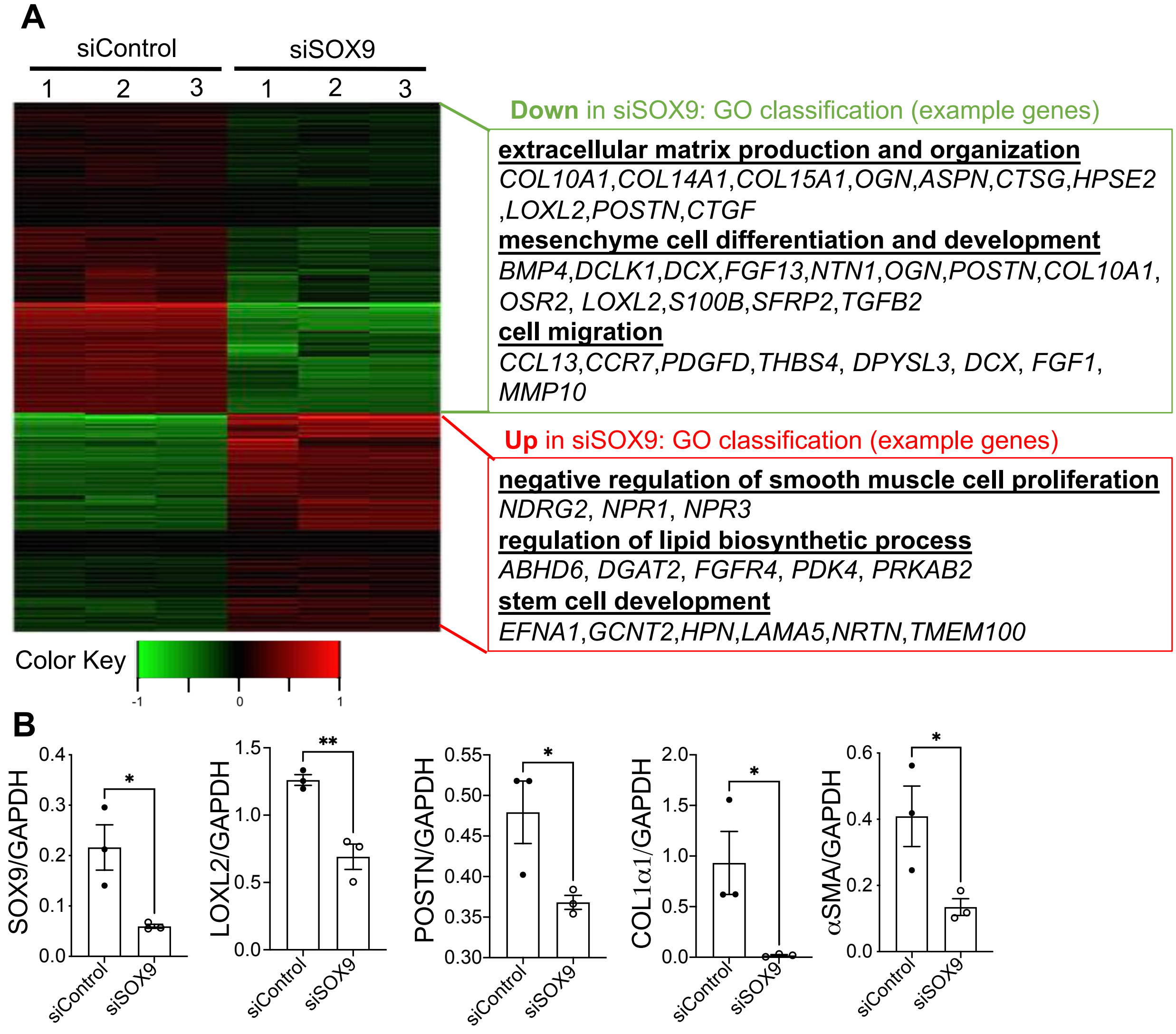


**Supplemental Figure 4. SOX9 is a positive regulator of several pro-fibrotic genes in IPF.** (A) Heat map of differentially expressed genes at least 1.5-fold with the knockdown of SOX9 in IPF fibroblasts by RNA-sequencing (n = 3). Function enrichment analysis of differentially expressed genes by SOX9 in IPF. (B) Quantification of immunoblots of SOX9, ACTA2, COL1α1, LOXL2, and POSTN in the lysates of IPF fibroblasts treated with either control or *SOX9*-specific siRNA for 72 hrs (**P <0.005, *P <0.05 n = 3, unpaired t-test).

**Supplemental Figure 5**

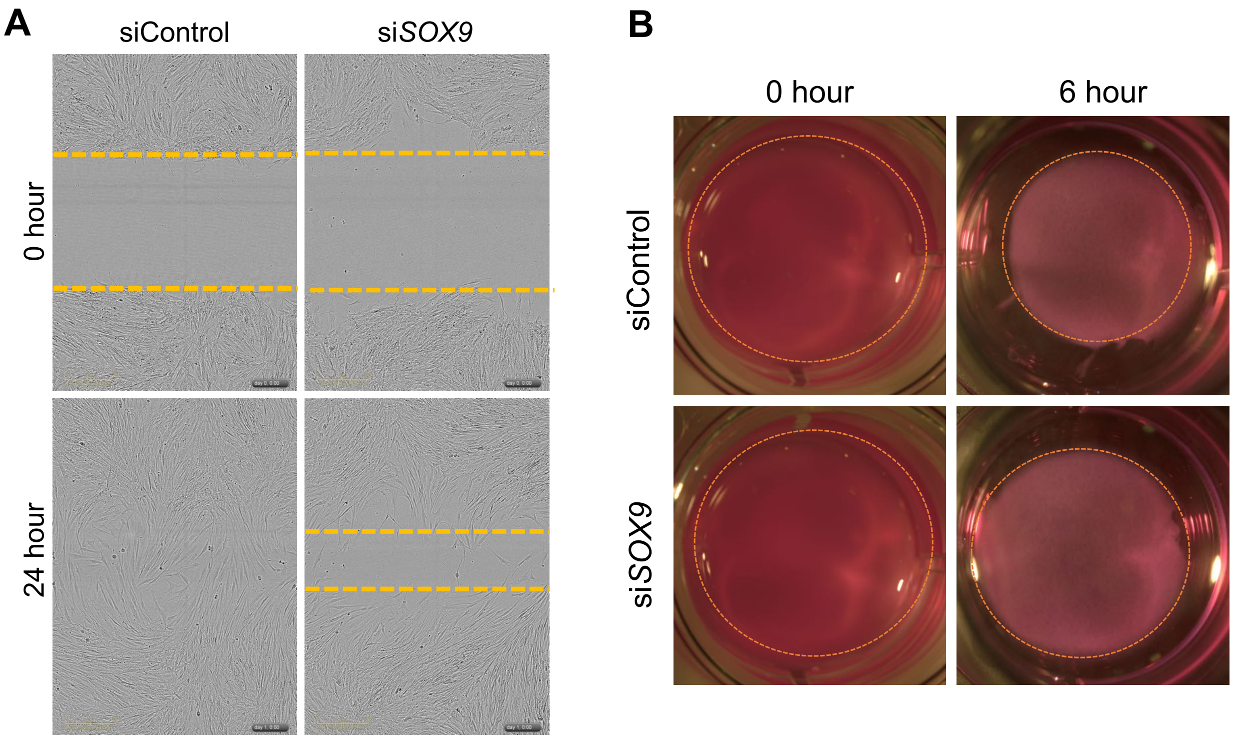


**Supplemental Figure 5. SOX9 promotes fibroblast migration and contraction of collagen gels.** (A) Representative bright-field images of 3D scratch assay performed on IPF fibroblasts treated with either control or *SOX9* siRNA for 48 hrs and imaged for 24 hrs after cells scratched. The growing edges are colored in orange. Images are obtained at 10x magnification with scale bar 300 μm. (B) IPF fibroblasts are treated with control or *SOX9*-specific siRNA for 72 hrs and seeded them into collagen gels for 6 hrs. Representative images of collagen gels at 0 and 6 hrs that marked using a dotted circle.

**Supplemental Figure 6**

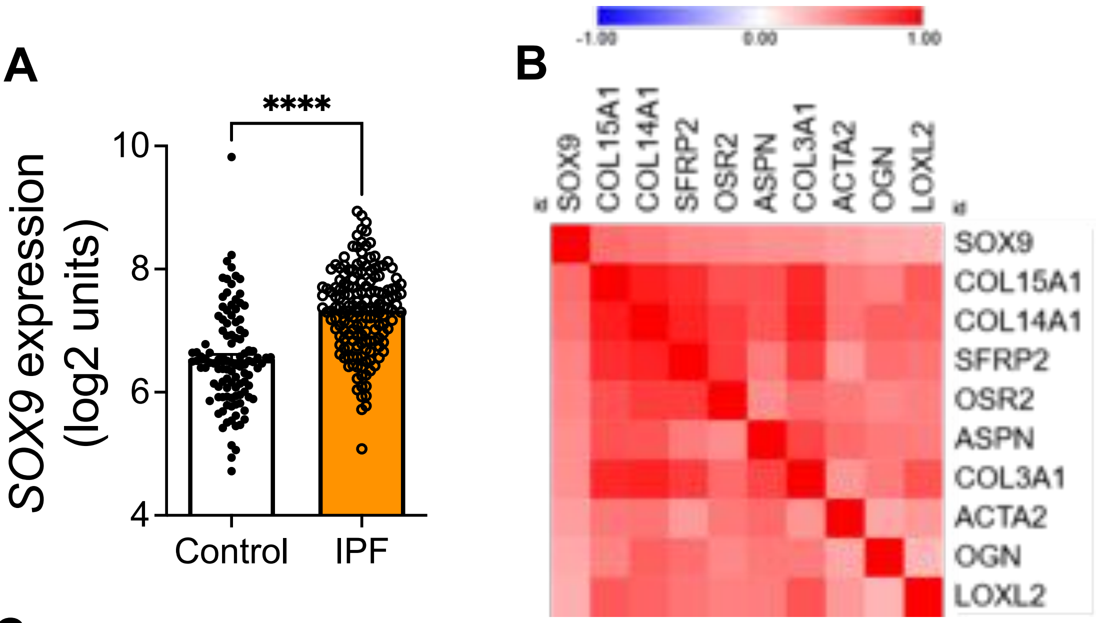


C

| Gene | Cor_FVC_IPF | Cor_DLCO_IPF | logFC |
|---|---|---|---|
| *SOX9* | -0.38 | -0.44 | 0.79 |
| *COL3A1* | -0.55 | -0.58 | 2.13 |
| *COL15A1* | -0.55 | -0.59 | 1.74 |
| *COL14A1* | -0.54 | -0.64 | 1.84 |
| *SFRP2* | -0.53 | -0.58 | 2.64 |
| *ASPN* | -0.44 | -0.48 | 1.37 |
| *OSR2* | -0.38 | -0.5 | 1.04 |
| *LOXL2* | -0.31 | -0.34 | 0.48 |
| *ACTA2* | -0.3 | -0.38 | 0.58 |
| *OGN* | -0.24 | -0.3 | 0.75 |

**Supplemental Figure 6. Correlation analysis of SOX9 and its target gene expression in the LGRC cohort.** (A) *SOX9* transcripts expression in control and IPF from the LGRC cohort (****P <0.00005, n = 108-160, unpaired t-test). (B) Heatmap of Pearson correlation matrix of *SOX9* expression and its target genes (*COL15A1*, *COL14A1*, *SFRP2*, *OSR2*, *ASPN*, *COL3A1*, *ACTA2*, *OGN*, and *LOXL2*. Dark red denotes higher correlation. (C) Expression of *SOX9* and its target genes and their Pearson correlation with lung function (FVC and DLCO) in the LGRC cohort. Green denotes anti-correlation and red denotes the log 2-fold change (p-value FDR B&H <0.05).

**Supplemental Tables:**

**Table S1.** The list of IPF genes that are up or down regulated with the knockdown of SOX9 in lung fibroblasts isolated from IPF lungs.

| Gene ID | Symbol | Annotation | log2 FC | pval | padj (FDR) |
|---|---|---|---|---|---|
| 2843 | GPR20 | IPF-DN SOX9KD-UP | 3.46 | 0 | 0 |
| 4902 | NRTN | IPF-DN SOX9KD-UP | 3.31 | 0 | 0 |
| 3249 | HPN | IPF-DN SOX9KD-UP | 2.58 | 0 | 0 |
| 478 | ATP1A3 | IPF-DN SOX9KD-UP | 1.9 | 0 | 0 |
| 1551 | CYP3A7 | IPF-DN SOX9KD-UP | 1.88 | 0 | 0 |
| 4778 | NFE2 | IPF-DN SOX9KD-UP | 1.7 | 0 | 0 |
| 6578 | SLCO2A1 | IPF-DN SOX9KD-UP | 1.68 | 0 | 0 |
| 50487 | PLA2G3 | IPF-DN SOX9KD-UP | 1.64 | 0 | 0.01 |
| 1942 | EFNA1 | IPF-DN SOX9KD-UP | 1.62 | 0 | 0 |
| 4881 | NPR1 | IPF-DN SOX9KD-UP | 1.6 | 0 | 0 |
| 168544 | ZNF467 | IPF-DN SOX9KD-UP | 1.56 | 0 | 0 |
| 5166 | PDK4 | IPF-DN SOX9KD-UP | 1.56 | 0 | 0 |
| 140730 | RIMS4 | IPF-DN SOX9KD-UP | 1.46 | 0 | 0.05 |
| 84649 | DGAT2 | IPF-DN SOX9KD-UP | 1.44 | 0 | 0 |
| 93010 | B3GNT7 | IPF-DN SOX9KD-UP | 1.44 | 0 | 0 |
| 885 | CCK | IPF-DN SOX9KD-UP | 1.41 | 0 | 0 |
| 1131 | CHRM3 | IPF-DN SOX9KD-UP | 1.4 | 0 | 0.05 |
| 80323 | CCDC68 | IPF-DN SOX9KD-UP | 1.35 | 0 | 0 |
| 9806 | SPOCK2 | IPF-DN SOX9KD-UP | 1.32 | 0 | 0 |
| 8839 | WISP2 | IPF-DN SOX9KD-UP | 1.31 | 0 | 0 |
| 5126 | PCSK2 | IPF-DN SOX9KD-UP | 1.3 | 0 | 0 |
| 117248 | GALNT15 | IPF-DN SOX9KD-UP | 1.25 | 0 | 0 |
| 1850 | DUSP8 | IPF-DN SOX9KD-UP | 1.23 | 0 | 0 |
| 84879 | MFSD2A | IPF-DN SOX9KD-UP | 1.2 | 0 | 0 |
| 79083 | MLPH | IPF-DN SOX9KD-UP | 1.13 | 0 | 0 |
| 55640 | FLVCR2 | IPF-DN SOX9KD-UP | 1.11 | 0 | 0 |
| 83733 | SLC25A18 | IPF-DN SOX9KD-UP | 1.08 | 0 | 0.02 |
| 5362 | PLXNA2 | IPF-DN SOX9KD-UP | 1.07 | 0 | 0 |
| 1E+08 | UPK3B | IPF-DN SOX9KD-UP | 1.04 | 0 | 0.01 |
| 1577 | CYP3A5 | IPF-DN SOX9KD-UP | 1.03 | 0 | 0 |
| 1318 | SLC31A2 | IPF-DN SOX9KD-UP | 1.01 | 0 | 0 |
| 140738 | TMEM37 | IPF-DN SOX9KD-UP | 1 | 0 | 0 |
| 745 | MYRF | IPF-DN SOX9KD-UP | 0.99 | 0 | 0 |
| 26112 | CCDC69 | IPF-DN SOX9KD-UP | 0.96 | 0 | 0 |

| | | | | | |
|---|---|---|---|---|---|
| 2651 | GCNT2 | IPF-DN SOX9KD-UP | 0.95 | 0 | 0 |
| 2264 | FGFR4 | IPF-DN SOX9KD-UP | 0.95 | 0 | 0 |
| 84612 | PARD6B | IPF-DN SOX9KD-UP | 0.95 | 0 | 0.05 |
| 4883 | NPR3 | IPF-DN SOX9KD-UP | 0.94 | 0 | 0 |
| 5787 | PTPRB | IPF-DN SOX9KD-UP | 0.93 | 0 | 0 |
| 79148 | MMP28 | IPF-DN SOX9KD-UP | 0.93 | 0 | 0 |
| 256691 | MAMDC2 | IPF-DN SOX9KD-UP | 0.87 | 0 | 0 |
| 11185 | INMT | IPF-DN SOX9KD-UP | 0.87 | 0 | 0 |
| 9056 | SLC7A7 | IPF-DN SOX9KD-UP | 0.86 | 0 | 0 |
| 1525 | CXADR | IPF-DN SOX9KD-UP | 0.85 | 0 | 0.01 |
| 57213 | SPRYD7 | IPF-DN SOX9KD-UP | 0.85 | 0 | 0 |
| 133522 | PPARGC1B | IPF-DN SOX9KD-UP | 0.84 | 0 | 0 |
| 80223 | RAB11FIP1 | IPF-DN SOX9KD-UP | 0.83 | 0 | 0 |
| 30061 | SLC40A1 | IPF-DN SOX9KD-UP | 0.79 | 0 | 0 |
| 57447 | NDRG2 | IPF-DN SOX9KD-UP | 0.79 | 0 | 0 |
| 256949 | KANK3 | IPF-DN SOX9KD-UP | 0.76 | 0 | 0.02 |
| 5565 | PRKAB2 | IPF-DN SOX9KD-UP | 0.76 | 0 | 0 |
| 3911 | LAMA5 | IPF-DN SOX9KD-UP | 0.72 | 0 | 0 |
| 56971 | CEACAM19 | IPF-DN SOX9KD-UP | 0.71 | 0 | 0 |
| 55273 | TMEM100 | IPF-DN SOX9KD-UP | 0.69 | 0 | 0 |
| 6272 | SORT1 | IPF-DN SOX9KD-UP | 0.69 | 0 | 0 |
| 2281 | FKBP1B | IPF-DN SOX9KD-UP | 0.69 | 0 | 0 |
| 57406 | ABHD6 | IPF-DN SOX9KD-UP | 0.68 | 0 | 0 |
| 1066 | CES1 | IPF-DN SOX9KD-UP | 0.67 | 0 | 0 |
| 6641 | SNTB1 | IPF-DN SOX9KD-UP | 0.67 | 0 | 0 |
| 8639 | AOC3 | IPF-DN SOX9KD-UP | 0.67 | 0 | 0 |
| 7732 | RNF112 | IPF-DN SOX9KD-UP | 0.65 | 0 | 0 |
| 140688 | NOL4L | IPF-DN SOX9KD-UP | 0.64 | 0 | 0 |
| 387758 | FIBIN | IPF-DN SOX9KD-UP | 0.63 | 0 | 0 |
| 6809 | STX3 | IPF-DN SOX9KD-UP | 0.62 | 0 | 0 |
| 124 | ADH1A | IPF-DN SOX9KD-UP | 0.62 | 0 | 0 |
| 51162 | EGFL7 | IPF-DN SOX9KD-UP | 0.62 | 0 | 0 |
| 2013 | EMP2 | IPF-DN SOX9KD-UP | 0.6 | 0 | 0 |
| 8459 | TPST2 | IPF-DN SOX9KD-UP | 0.6 | 0 | 0 |
| 1193 | CLIC2 | IPF-DN SOX9KD-UP | 0.6 | 0 | 0 |
| 2159 | F10 | IPF-DN SOX9KD-UP | 0.6 | 0 | 0 |
| 7079 | TIMP4 | IPF-DN SOX9KD-UP | 0.59 | 0 | 0 |
| 1809 | DPYSL3 | IPF-UP SOX9KD-DN | -0.59 | 0 | 0 |
| 57569 | ARHGAP20 | IPF-UP SOX9KD-DN | -0.61 | 0 | 0 |

| | | | | | |
|---|---|---|---|---|---|
| 652 | BMP4 | IPF-UP SOX9KD-DN | -0.62 | 0 | 0 |
| 353500 | BMP8A | IPF-UP SOX9KD-DN | -0.62 | 0 | 0.05 |
| 112476 | PRRT2 | IPF-UP SOX9KD-DN | -0.65 | 0 | 0 |
| 192683 | SCAMP5 | IPF-UP SOX9KD-DN | -0.65 | 0 | 0.02 |
| 2246 | FGF1 | IPF-UP SOX9KD-DN | -0.65 | 0 | 0 |
| 971 | CD72 | IPF-UP SOX9KD-DN | -0.65 | 0 | 0.01 |
| 6285 | S100B | IPF-UP SOX9KD-DN | -0.66 | 0 | 0.03 |
| 284454 | LOC284454 | IPF-UP SOX9KD-DN | -0.66 | 0 | 0 |
| 4319 | MMP10 | IPF-UP SOX9KD-DN | -0.68 | 0 | 0 |
| 1306 | COL15A1 | IPF-UP SOX9KD-DN | -0.69 | 0 | 0 |
| 80206 | FHOD3 | IPF-UP SOX9KD-DN | -0.69 | 0 | 0 |
| 5799 | PTPRN2 | IPF-UP SOX9KD-DN | -0.7 | 0 | 0 |
| 8111 | GPR68 | IPF-UP SOX9KD-DN | -0.7 | 0 | 0 |
| 220323 | OAF | IPF-UP SOX9KD-DN | -0.71 | 0 | 0 |
| 160518 | DENND5B | IPF-UP SOX9KD-DN | -0.73 | 0 | 0 |
| 9627 | SNCAIP | IPF-UP SOX9KD-DN | -0.73 | 0 | 0 |
| 11075 | STMN2 | IPF-UP SOX9KD-DN | -0.74 | 0 | 0.01 |
| 7171 | TPM4 | IPF-UP SOX9KD-DN | -0.74 | 0 | 0 |
| 121601 | ANO4 | IPF-UP SOX9KD-DN | -0.75 | 0 | 0 |
| 3512 | JCHAIN | IPF-UP SOX9KD-DN | -0.76 | 0 | 0 |
| 2258 | FGF13 | IPF-UP SOX9KD-DN | -0.76 | 0 | 0 |
| 5055 | SERPINB2 | IPF-UP SOX9KD-DN | -0.77 | 0 | 0 |
| 24141 | LAMP5 | IPF-UP SOX9KD-DN | -0.82 | 0 | 0 |
| 124975 | GGT6 | IPF-UP SOX9KD-DN | -0.83 | 0 | 0.03 |
| 57484 | RNF150 | IPF-UP SOX9KD-DN | -0.83 | 0 | 0 |
| 26140 | TTLL3 | IPF-UP SOX9KD-DN | -0.85 | 0 | 0 |
| 5743 | PTGS2 | IPF-UP SOX9KD-DN | -0.86 | 0 | 0 |
| 167838 | TXLNB | IPF-UP SOX9KD-DN | -0.87 | 0 | 0 |
| 7060 | THBS4 | IPF-UP SOX9KD-DN | -0.88 | 0 | 0 |
| 7373 | COL14A1 | IPF-UP SOX9KD-DN | -0.89 | 0 | 0 |
| 1300 | COL10A1 | IPF-UP SOX9KD-DN | -0.89 | 0 | 0 |
| 83758 | RBP5 | IPF-UP SOX9KD-DN | -0.9 | 0 | 0 |
| 9899 | SV2B | IPF-UP SOX9KD-DN | -0.91 | 0 | 0.04 |
| 4969 | OGN | IPF-UP SOX9KD-DN | -0.91 | 0 | 0 |
| 6357 | CCL13 | IPF-UP SOX9KD-DN | -0.92 | 0 | 0.01 |
| 55384 | MEG3 | IPF-UP SOX9KD-DN | -0.92 | 0 | 0 |
| 83690 | CRISPLD1 | IPF-UP SOX9KD-DN | -0.93 | 0 | 0.04 |
| 399726 | CASC10 | IPF-UP SOX9KD-DN | -0.94 | 0 | 0 |
| 220 | ALDH1A3 | IPF-UP SOX9KD-DN | -0.94 | 0 | 0 |

| | | | | | |
|---|---|---|---|---|---|
| 7042 | TGFB2 | IPF-UP SOX9KD-DN | -0.95 | 0 | 0 |
| 116039 | OSR2 | IPF-UP SOX9KD-DN | -0.96 | 0 | 0 |
| 2444 | FRK | IPF-UP SOX9KD-DN | -0.96 | 0 | 0 |
| 54829 | ASPN | IPF-UP SOX9KD-DN | -0.99 | 0 | 0 |
| 84624 | FNDC1 | IPF-UP SOX9KD-DN | -0.99 | 0 | 0 |
| 9308 | CD83 | IPF-UP SOX9KD-DN | -1.02 | 0 | 0 |
| 8000 | PSCA | IPF-UP SOX9KD-DN | -1.06 | 0 | 0.01 |
| 624 | BDKRB2 | IPF-UP SOX9KD-DN | -1.15 | 0 | 0 |
| 9201 | DCLK1 | IPF-UP SOX9KD-DN | -1.16 | 0 | 0 |
| 10631 | POSTN | IPF-UP SOX9KD-DN | -1.19 | 0 | 0 |
| 56475 | RPRM | IPF-UP SOX9KD-DN | -1.2 | 0 | 0 |
| 79605 | PGBD5 | IPF-UP SOX9KD-DN | -1.21 | 0 | 0 |
| 6423 | SFRP2 | IPF-UP SOX9KD-DN | -1.23 | 0 | 0.04 |
| 148979 | GLIS1 | IPF-UP SOX9KD-DN | -1.23 | 0 | 0 |
| 83643 | CCDC3 | IPF-UP SOX9KD-DN | -1.24 | 0 | 0 |
| 26166 | RGS22 | IPF-UP SOX9KD-DN | -1.26 | 0 | 0 |
| 53833 | IL20RB | IPF-UP SOX9KD-DN | -1.29 | 0 | 0 |
| 1E+08 | CD24 | IPF-UP SOX9KD-DN | -1.33 | 0 | 0 |
| 80310 | PDGFD | IPF-UP SOX9KD-DN | -1.37 | 0 | 0 |
| 4017 | LOXL2 | IPF-UP SOX9KD-DN | -1.4 | 0 | 0 |
| 5790 | PTPRCAP | IPF-UP SOX9KD-DN | -1.41 | 0 | 0 |
| 218 | ALDH3A1 | IPF-UP SOX9KD-DN | -1.42 | 0 | 0.02 |
| 6489 | ST8SIA1 | IPF-UP SOX9KD-DN | -1.42 | 0 | 0 |
| 60495 | HPSE2 | IPF-UP SOX9KD-DN | -1.46 | 0 | 0 |
| 1236 | CCR7 | IPF-UP SOX9KD-DN | -1.47 | 0 | 0 |
| 7634 | ZNF80 | IPF-UP SOX9KD-DN | -1.49 | 0 | 0.05 |
| 1734 | DIO2 | IPF-UP SOX9KD-DN | -1.58 | 0 | 0 |
| 1511 | CTSG | IPF-UP SOX9KD-DN | -1.6 | 0 | 0.01 |
| 6092 | ROBO2 | IPF-UP SOX9KD-DN | -1.67 | 0 | 0 |
| 9423 | NTN1 | IPF-UP SOX9KD-DN | -1.67 | 0 | 0 |
| 6328 | SCN3A | IPF-UP SOX9KD-DN | -1.91 | 0 | 0 |
| 338645 | LUZP2 | IPF-UP SOX9KD-DN | -1.96 | 0 | 0 |
| 1641 | DCX | IPF-UP SOX9KD-DN | -2.07 | 0 | 0.01 |

**Table S2**. The list of human RT-PCR primers used in the study

| Gene Symbol | Forward primer | Reverse primer |
|---|---|---|
| *ACTA2* | GCTTTCAGCTTCCCTGAACA | GGAGCTGCTTCACAGGATTC |
| *ASPN* | GGTGGATAACTTCTACTTTTAGGAGGA | AAGAAGGGTTTGGCAGAGC |
| *BCL-2L2* | TGGATGGTGGCCTACCTG | CGTCCCCGTATAGAGCTGTG |
| *BCL-XL* | GCCACTTACCTGAATGACCAC | TGCTGCATTGTTCCCATAGA |
| *COL1A1* | GGGATTCCCTGGACCTAAAG | GGAACACCTCGCTCTCCA |
| *COL14A1* | GGGGGAAGACTGAGGAGGT | CAATAGTGAAACTTCATACTCCGTACC |
| *COL15A1* | TCCAGGGGTCATTATGCAG | AAAATGGCTCCTTTGATGTTAATC |
| *COL3A1* | TGGTGGTAAAGGCGAAATG | AGTCCAGGAGCACCATTAGC |
| *CTGF* | TTCCAGAGCAGCTGCAAGTACCA | TTGTCATTGGTAACCCGGGTGGA |
| *DPYSL3* | GGTCCCGCGGCAGAAATAC | GGCATCGAAATCCAGCGTCT |
| *FGF1* | CACATTCAGCTGCAGCTCAG | TGCTTTCTGGCCATAGTGAGTC |
| *LOXL2* | GGAGAGGACATACAATACCAAAGTGT | CCATGGAGAATGGCCAGTAG |
| *MMP10* | TGGACAGAAGATGCATCAGG | CTTCAGTGTTGGCTGAGTGAA |
| *OGN* | AAAACTTCAAAACAGCTACAACGAC | TGGCTTTATCAGAGGCACAA |
| *OSR2* | ATGACCTTTCCAAGCTGTGC | CCAGTGAGACAACAGCACGTA |
| *POSTN* | TGAGAATGGAAGGAATGAAAGG | GCCCAGAGTGCCATAAACAT |
| *SFRP2* | GCTAGCAGCGACCACCTC | TTTTTGCAGGCTTCACATACC |
| *SOX9* | GTACCCGCACTTGCACAAC | TCTCGCTCTCGTTCAGAAGTC |
| *TGFB2* | ACAACACCCTCTGGCTCAGT | TAGAAAGTGGGCGGGATG |
| *THBS4* | CCTGAGACCATTGAATTGAGG | ACCAGCTTCAGCTCTTCCAA |
| *WT1* | AGCTGTCCCACTTACAGATGC | CCTTGAAGTCACACTGGTATGG |
| *β-ACTIN* | CCAACCGCGAGAAGATGA | CCAGAGGCGTACAGGGATAG |

**Table S3**. The list of mouse RT-PCR primers used in the study

| Gene Symbol | Forward primer | Reverse primer |
|---|---|---|
| *Acta2* | TGACGCTGAAGTATCCGATAGA | CGAAGCTCGTTATAGAAAGAGTGG |
| *Col1a1* | CATGTTCAGCTTTGTGGACCT | GCAGCTGACTTCAGGGATGT |
| *Col3a1* | CTCCTGGTGAGCGAGGAC | GACCAGGTTGCCCATCACT |
| *Col5a1* | CTACATCCGTGCCCTGGT | CCAGCACCGTCTTCTGGTAG |
| *Ctgf* | TGACCTGGAGGAAAACATTAAGA | AGCCCTGTATGTCTTCACACTG |
| *Fn1* | CGGAGAGAGTGCCCCTACTA | CGATATTGGTGAATCGCAGA |
| *Hprt* | GCCCTTGACTATAATGAGTACTTCAGG | TTCAACTTGCGCTCATCTTAGG |
| *Il13* | CCTCTGACCCTTAAGGAGCTTAT | CGTTGCACAGGGGAGTCT |
| *Il17* | CAGGGAGAGCTTCATCTGTGT | GCTGAGCTTTGAGGGATGAT |
| *Il6* | GCTACCAAACTGGATATAATCAGGA | CCAGGTAGCTATGGTACTCCAGAA |
| *Loxl2* | GAGCTTTTCTTCTGGGCAAC | CTCCATCCTTGTCCTGTGCT |
| *Mmp7* | TAATTGGCTTCGCAAGGAGA | AAGGCATGACCTAGAGTGTTCC |
| *Postn* | AAGCTGCGGCAAGACAAG | TCAAATCTGCAGCTTCAAGG |
| *Sox9* | GCAAAGTTGATCTGAAGCGAGAGGG | GATGTCCACGTCGCGGAAGTCGATG |
| *TGFb1* | GAGCTGCTTATCCCAGATTCA | GGCAGTGGAGACGTCAGATT |

**Table S4**. List of antibodies and their dilutions used for immunostaining

| Antibody | Dilution | | | Catalogue# | Company |
|---|---|---|---|---|---|
| | IHC | IF | WB | | |
| ACTA2 | 1:20000 | 1:2000 | 1:20000 | A5228 | Sigma |
| AKT | | | 1:1000 | 4685 | CST |
| COL1α1 | | | 1:1000 | 91144 | CST |
| COL1α1 | | | 1:500 | Sc-25974 | Santa Cruz Biotechnology |
| ERK | | | 1:1000 | 9102 | CST |
| FN1 | | | 1:500 | Sc-9068 | Santa Cruz Biotechnology |
| GAPDH | | | 1:2000 | A300-641 | Bethyl Laboratories |
| LOXL2 | | | 1:2000 | ab96233 | Abcam |
| p-AKT | | | 1:1000 | 9271 | CST |
| p-ERK | | | 1:1000 | 9101 | CST |
| POSTN | | | 1:1000 | NBP1-30042 | Novus Biologics |
| SOX9 | 1:1000 | 1:100 | 1:1000 | ab185966 | Abcam |
| VIM | | 1:50 | | Sc-7557 | Santa Cruz Biotechnology |
| WT1 | | | 1:500 | 12609-1-AP | Proteintech |
